# Hunting before herding: A zooarchaeological and stable isotopic study of suids (*Sus* sp.) at Hardinxveld-Giessendam, the Netherlands (5450–4250 cal BC)

**DOI:** 10.1371/journal.pone.0262557

**Published:** 2022-02-02

**Authors:** Nathalie Ø. Brusgaard, Michael W. Dee, Merita Dreshaj, Jolijn Erven, Youri van den Hurk, Daan Raemaekers, Canan Çakırlar

**Affiliations:** 1 Groningen Institute of Archaeology, University of Groningen, Groningen, The Netherlands; 2 Centre for Isotope Research, University of Groningen, Groningen, The Netherlands; Senckenberg Gesellschaft fur Naturforschung, GERMANY

## Abstract

Suids (*Sus* sp.) played a crucial role in the transition to farming in northern Europe and, like in many regions, in the Netherlands pig husbandry became an important subsistence activity at Neolithic sites. Yet little is known about wild boar palaeoecology and hunting in the Late Mesolithic Netherlands with which to contextualize this transition. This paper presents the first multi-proxy analysis of archaeological suid remains in the Netherlands. It explores human-suid interactions at the Swifterbant culture sites of Hardinxveld-Giessendam Polderweg and De Bruin (5450–4250 BC) through biometric analysis, estimation of age-at-death, and stable carbon and nitrogen isotope analysis. The results reveal targeted hunting of adult wild boar in the Late Mesolithic (5450–4850 BC), with a possible shift over time towards more juveniles. The wild boar in this period are demonstrated to be of comparably large size to contemporary northern European populations and exhibiting a wide range of dietary regimes. In the final occupational period (4450–4250 BC), small suids are present, possibly domestic pigs, but there is no evidence of pig management. This study demonstrates that the nature of human-suid interactions varied over time, which may have been connected to changing environmental conditions, human mobility, and wild boar behaviour. This study also contributes the first biometric and dietary baseline for mid-Holocene wild boar in the Netherlands.

## Introduction

Suids (*Sus* sp.) were key to one the most pivotal developments in the history of human- and non-humankind: the transition to farming. In northern Europe, human-pig relationships at the advent of farming did not develop in a void but among pre-existing relationships between humans and wild boar (*Sus scrofa*). The arrival of the domestic pig (*Sus scrofa domesticus*) meant not the introduction of an entirely alien species but a domestic counterpart to the indigenous wild boar which humans had hunted and interacted with for millennia. This opens up a whole range of intriguing questions about the emergence of pig husbandry in northern Europe. Chief among these is the question of to what extent the indigenous wild boar played a role in this process, for example through their local domestication or hybridisation with domestic pigs. Genetic evidence in fact reveals that there was significant interbreeding between domestic pigs of Near Eastern descent and European wild boar in the millennia after the introduction of the former [1], further underscoring the importance of wild boar in the development of pig husbandry.

To understand this development, there have been substantial studies focused on disentangling early domestic pigs from wild boar in foraging and early farming societies in northern Europe, above the Linearbandkeramik zone of Central Europe [e.g. 2–4]. These have included looking at differences in size among suid populations, changes in harvesting profiles, and differences in diet and genetics [5]. One region, however, has been relatively absent from this debate: the ‘Swifterbant culture’ area. Sites belonging to the Swifterbant culture (ca. 5000–3400 BC) have been identified, based on its distinct material culture, in the Netherlands, Belgium, and Lower Saxony [6, 7]. In the Netherlands, Swifterbant sites include rich faunal assemblages that have had significant impact on our understanding of the transition to farming in northern Europe. Suids are well-represented, and typically more than any other large mammal, in these assemblages. However, studies of such suids have until now primarily concentrated on identifying wild boar versus domestic pigs, based on morphology and individual metrics [e.g. 8–10] [but see 11]. Few multi-proxy studies have been carried out and recent methodological advances in the zooarchaeology of suids have not yet been applied. Furthermore, a major setback to any analysis of the emergence of domesticated pigs in the Swifterbant area is that little is known about wild boar and wild boar hunting in Mesolithic Netherlands. Therefore, there is scant evidence with which to contextualise the identification of domestic pigs and emergent suid management strategies.

**Fig 1:**
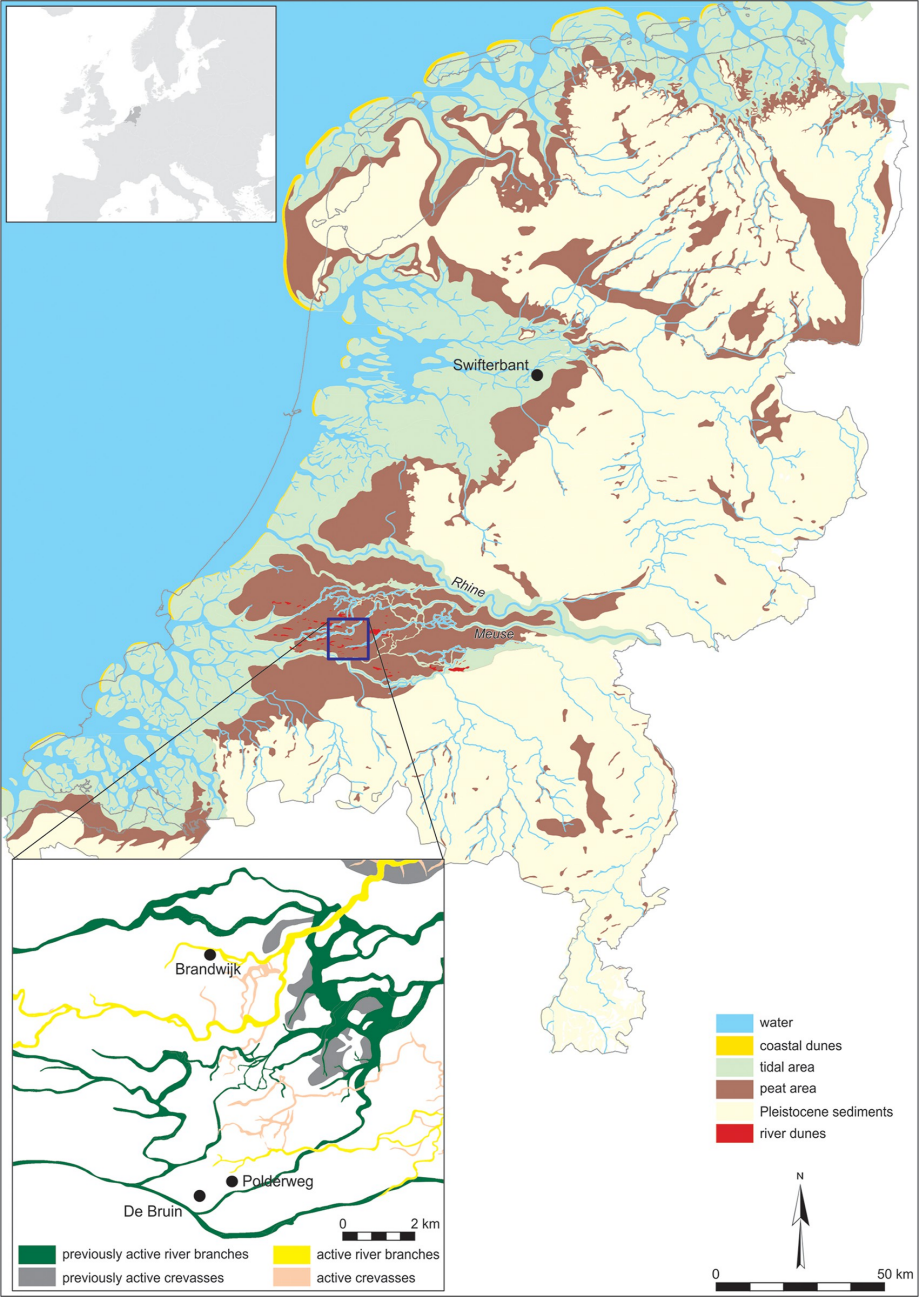
Palaeogeographical map of the Netherlands (3850 BC) showing the location of the Hardinxveld area (box) and the eponymous site Swifterbant, and local palaeogeographical map (insert) showing the location of the sites Polderweg, De Bruin, and Brandwijk (after Vos et al. [15]).

In this paper, we address this lacuna with an exploration of human-suid interactions through subsistence practices at Hardinxveld-Giessendam Polderweg and Hardinxveld-Giessendam De Bruin (hereafter Polderweg and De Bruin). These Swifterbant culture sites were situated adjacently in the Rhine-Meuse delta and together their occupation spans the period 5450 to 4250 BC (Fig 1). They have yielded the largest assemblage of suid remains from the Late Mesolithic and Early Neolithic in the Netherlands. Polderweg and De Bruin therefore offer a unique opportunity to reconstruct human-suid interactions in forager societies over a long time span in the prelude to animal husbandry. Both Polderweg and De Bruin have extensive zooarchaeological reports [9, 12]. However, since their publication the assemblages have only been re-examined as part of wider zooarchaeological studies [13, 14]. Since then, new methods have been developed and others reinforced for the study of age, size, and diet of suids. Considering the importance of these assemblages, the time is ripe for a re-investigation.

In this paper, we re-examine the suid remains through biometric, age estimation, and stable carbon and nitrogen isotope analysis. With these methods we endeavour to shed light on suid size, harvesting profiles, and diet. These factors can provide insight into the beginnings of animal husbandry, when the eventual biological and economic changes this process brought about might not yet be clearly visible in the archaeological record. Equally, they can provide insights into wild boar and their relationship with humans prior to the transition to pig husbandry. Using these methods, we aim first to investigate wild boar palaeoecology and hunting strategy in the Late Mesolithic. Second, we endeavour to create a baseline of wild boar size and diet that can be used to further identify domesticated and managed pig populations. Third, we aim to uncover whether there is evidence for domesticated pigs towards the end of the occupation sequence. Through this multi-proxy study, this paper contributes new insights into human-suid interactions in the prelude to animal husbandry in northern Europe.

## The Hardinxveld-Giessendam sites

### Chronology

Polderweg (ca. 5450–4650 BC) and De Bruin (ca. 5400–4250 BC) are Swifterbant culture sites that were located on neighbouring river dunes, approximately 1 km apart. Both sites show stratified phases of occupation [16–18] (Table 1). The phases are separated by layers with few to no finds, suggesting either hiatuses in between occupation or periods of low-intensity occupation [17, 18]. The vast majority of finds from Polderweg belong to Period 1, whereas Period 2 is represented by just a small concentration of finds [17]. In contrast, most of De Bruin’s finds stem from Period 2. There is subsequently some activity at Polderweg in Period 3, again indicated by small artefact clusters, but no activity at De Bruin. Finally, after a hiatus, occupation recommenced at De Bruin during Period 4. By then rising water levels and continuous sedimentation of the surrounding area had submerged Polderweg, a fate that befell De Bruin by the end of the fifth millennium [17–19].

**Table 1:** Chronological developments at Polderweg and De Bruin.

Chronological Period	Polderweg (dates BC)	De Bruin (dates BC)	Features
Period 0	Pre-5450 *Phase 0*		A single human burial and pit that stratigraphically pre-date the first occupation phase at Polderweg.
Period 1	5450–5300 *Phase 1*	5400–5150 *Phase 1*	Seasonal winter camps; intensive use of Polderweg.
Period 2	5250–4800 *Phase 1/2*	5000–4850 *Phase 2*	Ceramics and occupation during multiple seasons at De Bruin.
			Polderweg occupation demonstrated by only five artefact concentrations.
Period 3	4850–4650 *Phase 2*		Ceramics at Polderweg. Occupation demonstrated by only one artefact concentration. No evidence for occupation at De Bruin.
Period 4		4450–4250	Domesticated sheep/goat at De Bruin.
		*Phase 3*	No evidence for occupation at Polderweg.

For ease of reference, we have labelled the chronological Phases Periods 0–4, which will be used throughout. The corresponding phases identified during the excavations are written in italics. For chronology see Dreshaj et al. (forthcoming). For detailed information on the features in each period see Louwe Kooijmans [20, 21].

This timespan shows first, seasonal broad-spectrum forager activity at both sites, then the introduction of Swifterbant pottery and multi-seasonal use, and lastly the appearance of domesticated sheep/goat in the last occupational period [9, 11, 12, 22–24]. Various lines of evidence point to the river dunes being used as a base, at least in the Late Mesolithic periods, consistently by the same groups [25]. The final period of occupation is contemporary with occupation at Swifterbant culture sites at nearby Brandwijk (ca 4600–3600 cal BC) and the eponymous Swifterbant (ca 4300–4000 cal BC), where there is zooarchaeological and genetic evidence of domestic (or at least managed) pigs [8, 10, 11, 26].

### Palaeoenvironment

The Polderweg and De Bruin sites were located within a marshy area of the Rhine-Meuse river delta. This freshwater landscape was characterised by marshland, slow meandering rivers, alder carr, and shallow eutrophic lakes [27–31]. The river dunes formed dry locations within this wetland environment. They were covered in an open, mixed deciduous forest of oak, ash, and lime trees, interspersed with herbs and shrubs. The surrounding wetland vegetation comprised alder carr punctuated by marsh and forb vegetation [27, 28, 31].

The faunal remains mirror the freshwater landscape, and the mix of marshland and open forest. The mammal remains are dominated by suids, Eurasian beaver (*Castor fiber*), and Eurasian otter (*Lutra lutra*) (Fig 1 in S1 Fig; Table 1 in S1 Dataset). Other species include cattle (*Bos* sp.) and cervids, including red deer (*Cervus elaphus*), roe deer (*Capreolus capreolus*), and elk (*Alces alces*), and a variety of other mammals occurring in small numbers. Remains of wetland bird and freshwater fish remains are plentiful at the sites [9, 12, 32, 33].

The environment shifted during Period 1, with an increase in marshland surrounding the river dunes and a halt in river flow [29, 30]. The vegetation on the dunes changed, with forest making way for more shrubs and marsh vegetation, probably also as a result of the increasingly moist environment [27, 28]. In Period 4 at De Bruin, the continuously rising water levels led to a further increase in marsh vegetation on the dune [27, 30].

### Subsistence

The zooarchaeological and archaeobotanical assemblages indicate that fishing, fowling, hunting, and gathering were core activities at the sites. Presence and absence of animal and plant species suggest that these were, at first, winter base camps, occupied from late autumn to early spring. A change occurred at De Bruin around 5000 BC (Period 2) whereupon there emerges zooarchaeological and archaeobotanical evidence for occupation in all seasons, suggesting the winter base camp was expanded to provide subsistence throughout the year, albeit perhaps not continuously [9, 12, 27, 28, 32, 33]. Polderweg did not yield enough remains from this period to compare this.

Throughout the sites’ occupation, hunting activities appear to have been focused primarily on suids, beavers, and otters. A change occurs in their relative abundance after Period 1, when the proportion of suids decreases and the proportion of beaver increases in Number of Fragment (NF) counts [9, 12]. Cattle (*Bos* sp.) remains are present in only very small numbers and, at Polderweg, only as artefacts, further underscoring the dominance of suids, beavers, and otters at these sites. This is typical of Dutch Swifterbant sites, where these three species tend to dominate the mammal assemblage, although there appears to have been an even more focused exploitation of beaver and otter at Hardinxveld than at other sites [10, 26].

The suid specimens are not dominated by any one part of the skeleton, suggesting that the carcasses were processed on-site. Among their remains, male canines are more numerous than female canines but most of these are artefacts, so the sex ratio is difficult to determine [9, 12].

The final occupation phase at De Bruin sees the appearance of domesticated livestock, notably small numbers of sheep/goat (*Ovis aries*/*Capra hircus*) remains [11]. The initial identification of a few domestic pig and cattle remains by Oversteegen et al. [9] is less secure, with a recent re-evaluation of the specimens in question not being able to confirm their domestic status using osteometry and survivorship data [11]. Dogs (*Canis familiaris*) are the only domestic animals found at Polderweg.

## Materials and methods

### Material

At both Polderweg and De Bruin, the same excavation and collection method was used. Faunal remains were collected by hand and by sieving through 4 mm mesh. Additionally, samples were taken, which were sieved through 2 mm mesh [34, 35]. All of the hand-collected faunal material from the site was documented and identified, along with 256 sieved samples randomly selected from areas with a high concentration of finds [9, 12]. This yielded a total of 4535 mammalian specimens that could be identified to species level for Polderweg and 2560 for De Bruin. The faunal material was analysed by Number of Fragments (NF) [9, 12]; in this study we maintain these counts for consistency.

For this study, to ensure the quality of the dataset, we re-assessed the 1055 identified suid remains from Polderweg and 387 from De Bruin. We updated identifications where necessary (Table 2 in S1 Dataset). To increase the sample size for the biometric analyses and age-estimation, we combed through the deselected, unidentified sieve material, yielding an additional 16 measurable suid remains from Polderweg and 35 from De Bruin (Table 2 in S1 Dataset).

Where possible, the analyses were carried out per period to investigate chronological developments. For Polderweg this was possible only to a very limited extent due to the small number of finds Periods 0, 2, and 3 (see Table 3 in S1 Dataset for sample size for each analysis).

### Biometric analysis

Distinguishing domestic pigs from wild boar is a well-known challenge [5, 36]. To alleviate the problem of small sample sizes, one of the most applied methods is Logarithmic Size Index analysis (LSI) of metric data. The LSI method measures the logarithmic difference of measurements on teeth or postcranial bones (fragmented and complete) and compares them to the same measurements on a ‘standard’ [37]. On the logarithmic scale, 0 represents the standard, and specimens that are smaller than the standard will scale negatively while specimens that are larger will scale positively. Recent studies continue to show that it is a better method than using direct assessment of single measurements [38]. Because suids are sexually dimorphic, size variation as shown by LSI can also be caused by differences in the sexual demography of an archaeological assemblage [39, 40]. This can be mitigated by comparing postcranial and dental LSI because sexual dimorphism is less pronounced in suid molars, and by using postcranial measurements less affected by sex [40, 41]. Conversely, the distribution of LSI values, for example a heavy skewness, can also provide insight into sex distribution, affording the opportunity to investigate possible selective hunting or herd management by humans [39–41]. This study represents one of the first applications of the LSI method in Dutch zooarchaeology.

We measured all measurable postcranial and dental elements from Polderweg and De Bruin. This yielded 75 postcranial measurements and 138 dental measurements (Tables 4–6 in S1 Dataset). The LSI analysis was carried out following Meadow [37] (base = 10). For the postcranial analysis, we used the wild boar standard established by Hongo and Meadow [42] (a female wild boar from Eastern Turkey). LSI values represent the average LSI of all measurements on one element, excluding length measurements except in the case of astragali and calcanea, following Meadow [37] and Albarella and Payne [43]. Scapula measurements were excluded to eliminate possible age bias [39, 43].

For the dental analysis, we used the wild boar standard established by Payne and Bull [40] (the mean of a wild boar population). LSI values were calculated per tooth and represent the average LSI of the width measurements on one tooth; length measurements are excluded and upper and lower teeth are analysed separately, following Zeder and Lemoine [41] and Meadow [37]. For each site and each period, the postcranial and dental data are Normally distributed (Table 7 in S1 Dataset). For this reason, we applied parametric statistical tests [following 38], using IBM SPSS Statistics 26.

To situate the Hardinxveld suids within the wider context, we compare our results with relevant assemblages in the greater region: Mesolithic assemblages from Scania, Sweden (ca 5800–4000 BC) [44], the Dutch Neolithic site of Schipluiden (ca 3600–3300 BC; only postcranial data) [45], and Durrington Walls (ca. 2800–2400 BC) [43]. Four outliers were excluded from Durrington Walls (all LSI smaller than -0.2).

### Age estimation

Recent methods for ageing suids using epiphyseal fusion and tooth wear provide means to create mortality profiles with greater resolution than previously possible [46, 47]. Mortality profiles offer insights into choices made by hunters and intensity of hunting, as well as–potentially–signs of a managed versus a hunted population [5, 48]. Changes in harvesting strategies, for example in sex and age of the animals, can signal a shift to domestication long before morphologically domestic animals are detectable in the archaeological record [49]. For example, an assemblage dominated by juvenile suids may indicate slaughtering of herded animals as soon as they reach their prime weight and, in particular, dominance of male juveniles may suggest herd management through culling of redundant animals [5, 39, 47, 49]. This can contrast with assemblages resulting from hunting, which may instead be dominated by older adult individuals that are targeted for more gain in terms of meat and fat [5, 48]. Other factors such as seasonality and sample size do need to be taken into account here [cf. 5].

To reconstruct harvesting profiles, we carried out age estimation using tooth wear/eruption and epiphyseal fusion. Tooth wear and eruption was scored and the subsequent survivorship percentage was calculated following Lemoine et al. [46] (System A) (Tables 8, 9 in S1 Dataset). The newly identified specimens were included in this analysis. The method is compatible with but offers advantages to the method of Grant [50]. Epiphyseal fusion data was obtained from the original faunal dataset and reassessed. Age classes and survivorship percentage were calculated following Zeder et al. (2015) (Tables 10, 11 in S1 Dataset).

### Stable isotope analysis

Stable isotope analysis has become a staple of zooarchaeological research, providing insight into past animal diet, mobility, and management [51, 52]. As a method for investigating human-animal interactions, the combination of osteological analysis with stable isotope analysis is particularly valuable because both provide detailed information on the life history of an individual animal and its relationship to humans [53]. Despite its demonstrated potential, however, stable isotope analysis has been used very sparingly in Dutch zooarchaeological studies with only a recent increase in its application [e.g. 54–56].

Stable carbon (δ^13^C) and nitrogen (δ^15^N) isotope values of bone collagen reflect the average ratios of the dietary protein consumed in the last years of an individual’s life and allow for the reconstruction of an individual’s diet, environment, and trophic level [57–59]. Terrestrial mammalian bone collagen δ^13^C and δ^15^N values are enriched 0–2‰ and 3–5‰, respectively, compared to the values of their diet [60–62]. Herbivores feeding on C_3_ plants in an open environment typically exhibit bone collagen δ^13^C values of between -22.5‰ and -18.5‰ [63]. C_4_ plants were absent in prehistoric Netherlands [64]. In a forested environment, a combination of deliberated factors cause more depleted δ^13^C values, known as the ‘canopy effect’ [65, 66]. In temperate forests, δ^13^C values lower than -22.5‰ are likely to be derived from a diet composed significantly of closed-canopy vegetation [67]. Freshwater or marine components in the diet also influence δ^13^C values. Humans subsisting primarily on marine foods exhibit elevated δ^13^C values of around -13‰, while freshwater sources may produce δ^13^C values as low as -24‰ but are generally more variable [60, 64, 68, 69]. Numerous factors besides trophic level can determine an individual’s δ^15^N values, including age and sex [70], suckling [57], nutritional stress [71], salinity of the environment [72], and manuring in the environment [73], although the latter is unlikely to be a factor in the period in question.

Suids are omnivores and opportunistic feeders, and therefore it is important to take into account a possible wide variety of dietary sources and factors in relation to their stable isotope ratios. Various studies have demonstrated the method’s potential for investigating both the emergence and nature of pig husbandry [3, 74–77]. Stable isotope analysis can help distinguish changes from the wild boar regime to a regime influenced by human management or interaction. For example, suids may consume more animal protein due to foraging on or being fed domestic waste that consists of meat or fish [3, 53, 78]. A study of human diet at Hardinxveld indicated a significant contribution from aquatic resources [69], which is supported by the high proportion of freshwater fish remains at the sites. This provides insight into possible domestic waste sources for suids. Similarly, anomalies in stable isotope values compared to known wild boar may signal early domestication processes, such as foddering or the confinement of suids to the open (human) habitat [76, 79]. To distinguish any changes due to the emergence of animal husbandry, it is of clear importance to first establish a baseline of wild boar δ^13^C and δ^15^N values [e.g. 78].

For our study, we selected primarily suid mandibles for sampling because this made it possible to establish age based on tooth wear. By controlling for the age of the individual in combination with consistently sampling one skeletal element, it is possible to establish that each specimen is a unique individual. Furthermore, it supplied extra depth to the life history of each individual, elucidating both diet and age. Where possible, we measured the sampled individuals to compare LSI to the stable isotope results.

Four cattle specimens from De Bruin were analysed to investigate further possible evidence of animal husbandry. The Polderweg cattle remains were artefacts and therefore not available for destructive analysis. The rest of the samples comprise wild faunal remains, analysed to establish an environmental and dietary baseline. The wild fauna selection was based on the most common taxa found at the Hardinxveld sites, a selection of different dietary regimes, and potential dietary resources for the suids. This includes red and roe deer, which provide baseline herbivorous δ^13^C and δ^15^N data and insight into potential environmental changes over time [80–82]. It includes the Eurasian otter, which has a diet comprising primarily freshwater fish [83, 84], allowing for comparison with potential suids feeding on human refuse at the sites. Eurasian beavers and mallards have been included as further background fauna, with beavers providing insight into δ^13^C and δ^15^N values of a generalist herbivore and possible changes in the local environment due to their small home ranges [85], and mallards shedding light on the generalist forager diet and changes in the aquatic environment [86].

In total, 70 samples were selected. To ensure sampling of unique individuals and thus avoid duplicate sampling, samples were selected from the same skeletal element for each species within each occupation phase or, when this was not possible, individuals were identified based on osteometrics and ageing. To prevent values influenced by suckling, samples were selected from individuals that could be determined to be older than weaning age based on tooth wear, fusion, or state of the bone.

Prior to sampling, the bones were documented thoroughly via photographs and 3D models using a DAVID 3D light scanner. Samples were not taken from sections of bone with visible taphonomic marks, such as cut marks, or from diagnostic parts of an element.

Samples of bone were cut using a Dremel or fractured, and then crushed. Collagen extraction and combustion took place at the Centre for Isotope Research (CIO) at the University of Groningen. Pretreatment and collagen extraction followed a modified Longin [87] protocol. The details of this procedure are outlined in Dee et al. [88]. Briefly, the bone was first demineralised in an HCl (4% w/vol) solution, replenished several times over 24 hours. This was followed by NaOH (1% w/vol) to remove humic acids, and then a final HCl (4% w/vol) treatment to eliminate any CO_2_ absorbed during the base phase. Each step was followed by a triplicate rinse in deionised and decarbonised water. The samples were then denatured to gelatin in deionised and decarbonised water (pH 3, 80°C, 18 hr) and dried.

Following pretreatment, 5–6 mg aliquots of extracted collagen were weighed out per sample. CIO quality control procedures require samples that yield less than 0.5% collagen to be rejected; this applied to 21 samples, of which seven were suids and two cattle. The rest were wild fauna specimens. A total of 49 samples were combusted.

The stable isotope ratios (δ^13^C and δ^15^N) were obtained via combustion in an elemental analyser (EA, Elementar Vario Isotope Cube) coupled to an Isotope Ratio Mass Spectrometer (IRMS, IsoPrime 100). The quality of collagen preservation was primarily assessed by the C:N ratio. The C:N range of 2.9–3.6 from DeNiro [58] is often cited as acceptable for collagen purity, but the CIO prefers to investigate results if they fall outside 3.1–3.3 [88], and is moving to rejection for all samples outside 2.9–3.5. It was only necessary to reject one sample based on this latter criterion.Each pretreatment batch included a secondary standard, with known isotope ratios, and a duplicate. The δ^13^C and δ^15^N data were obtained at the following 1σ precisions: δ^13^C = 0.15‰ and δ^15^N = 0.3‰.

The CIO stable isotope ratios are substantiated by a range of international references and inhouse controls. For δ13C, this is primarily caffeine (-38.2‰), and the IAEA oxalic acid (-17.60‰); and for δ^15^N, the same caffeine (-6.61‰) and CaN (caffeine with enriched nitrogen, +18.8‰). The controls include an in-house long-run collagen reference (δ^13^C = -19.87‰ and δ^15^N = +9.87‰). The percentages on combustion are substantiated by long-run measurements on said standards, and amount to ±0.4% for carbon and 0.2% for nitrogen on both caffeines, and ±0.3% and 0.9% on the collagen, respectively.

## Results

### Biometric analysis

**Fig 2:**
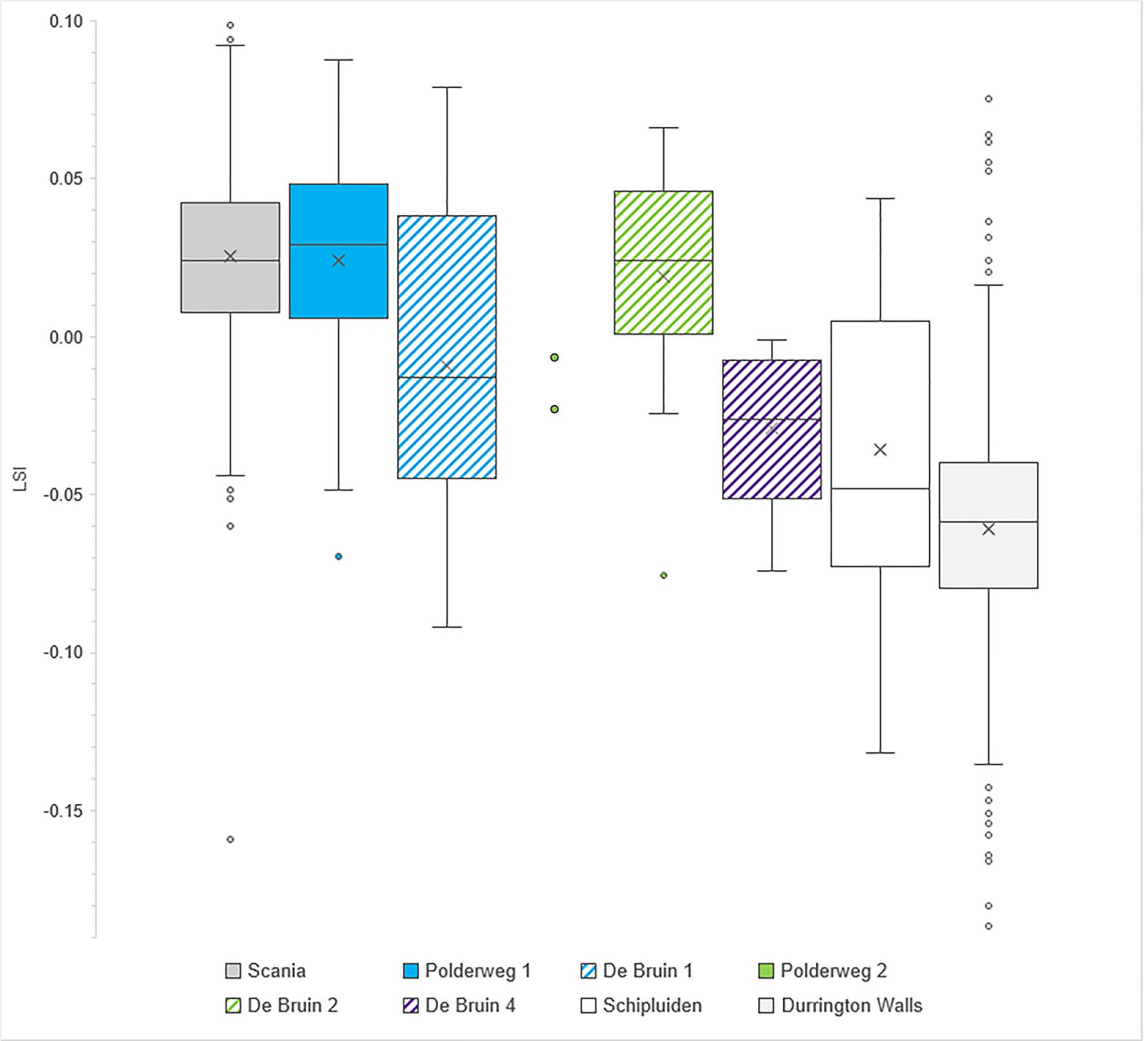
Boxplot comparing LSI values of fused postcranial elements from Scania (n = 324), Polderweg Period 1 (n = 38), De Bruin Period 1 (n = 11), Polderweg Period 2 (n = 2; shown as two points), De Bruin Period 2 (n = 18), De Bruin Period 4 (n = 6), Schipluiden (n = 17), and Durrington Walls (n = 843).

**Fig 3:**
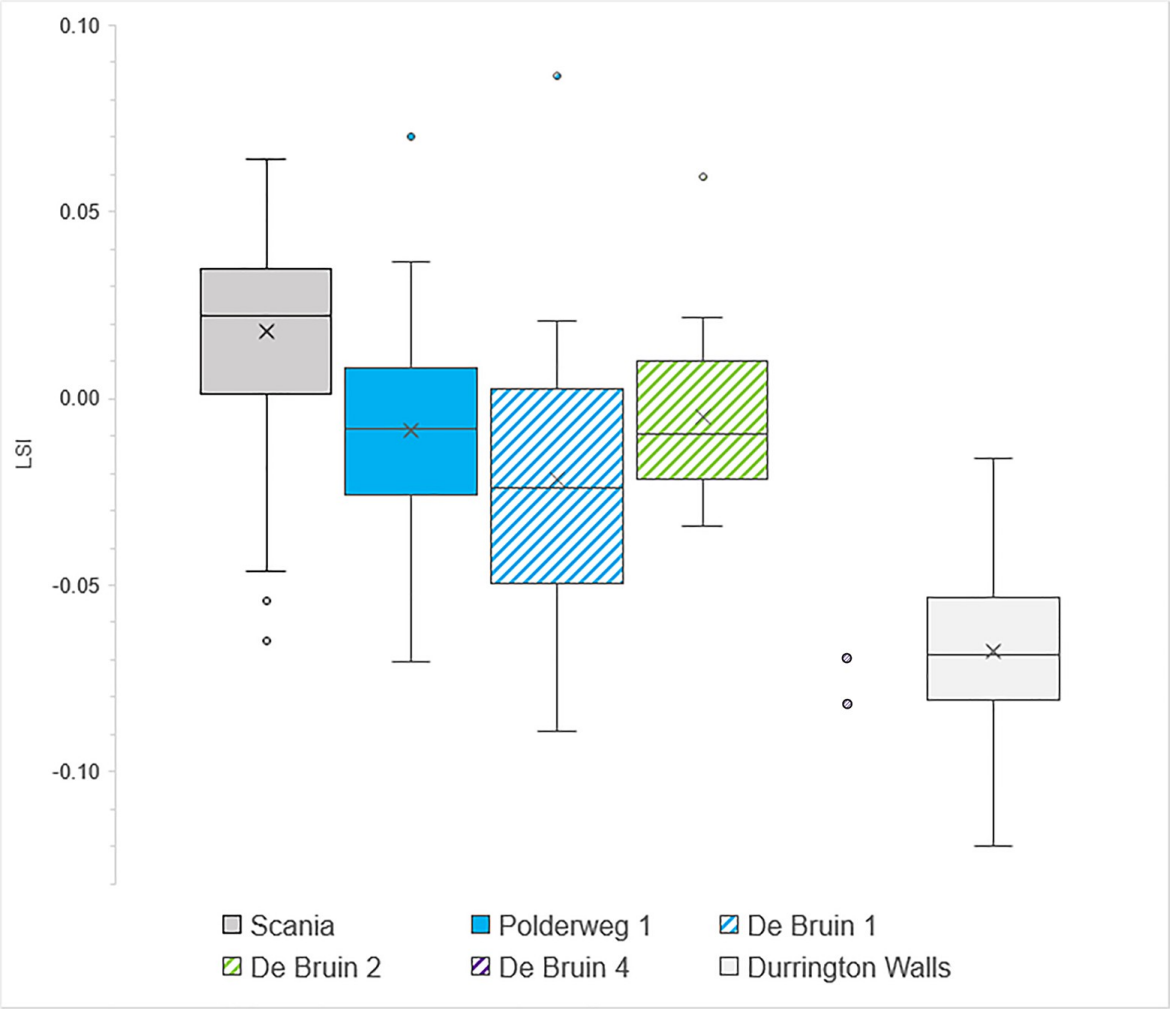
Boxplot comparing LSI values of fully erupted lower teeth from Scania (n = 145), Polderweg Period 1 (n = 49), De Bruin Period 1 (n = 15), De Bruin Period 2 (n = 14), De Bruin Period 4 (n = 2; shown as two points), and Durrington Walls (n = 334).

The logarithmic size indices of fused postcranial elements reveals that the suids from Period 1 scale differently at Polderweg and De Bruin (Fig 2; Fig 3 in S1 Fig). Polderweg specimens are significantly larger than specimens from this same period at De Bruin, with respective mean values of 0.021 and -0.012 (Independent T-test, p = 0.013; Tables 12, 13 in S1 Dataset). Specimens from the subsequent period at De Bruin, Period 2, scale larger than during the previous period (mean = 0.019) whereby the difference is approaching significance (Independent T-test, p = 0.052). De Bruin 2 scales close to Polderweg 1, with a mean difference of only 0.0014 (Independent T-test, p = 0.883). The Polderweg 1 and De Bruin 2 populations scale similarly to the large Scanian wild boar and slightly larger than the standard. The only two specimens from Period 2 at Polderweg have small values, comparable to De Bruin 1.

Period 4 –only represented at De Bruin–has the smallest elements with a mean of -0.032. They scale close to the suids from Schipluiden. They are significantly smaller than Period 2 of De Bruin (Independent T-test, p = 0.003). The difference with De Bruin 1 is not significant (Independent T-test, p = 0.327). The sample size is small, however; only six specimens could be measured from De Bruin 4. To compare the three periods at De Bruin to each other, an ANOVA test was carried out; it showed significant difference between the three periods (p = 0.013). The specimens from the last occupational phase scale very close to the suids at Schipluiden (mean = -0.036) but not as small as the domestic pig population at Durrington Walls. The smaller elements from De Bruin 1 and 4 are probably the reason why the De Bruin suids as a whole scale significantly smaller than the Polderweg suids as a whole (Independent T-test, p = 0.041).

**Fig 4:**
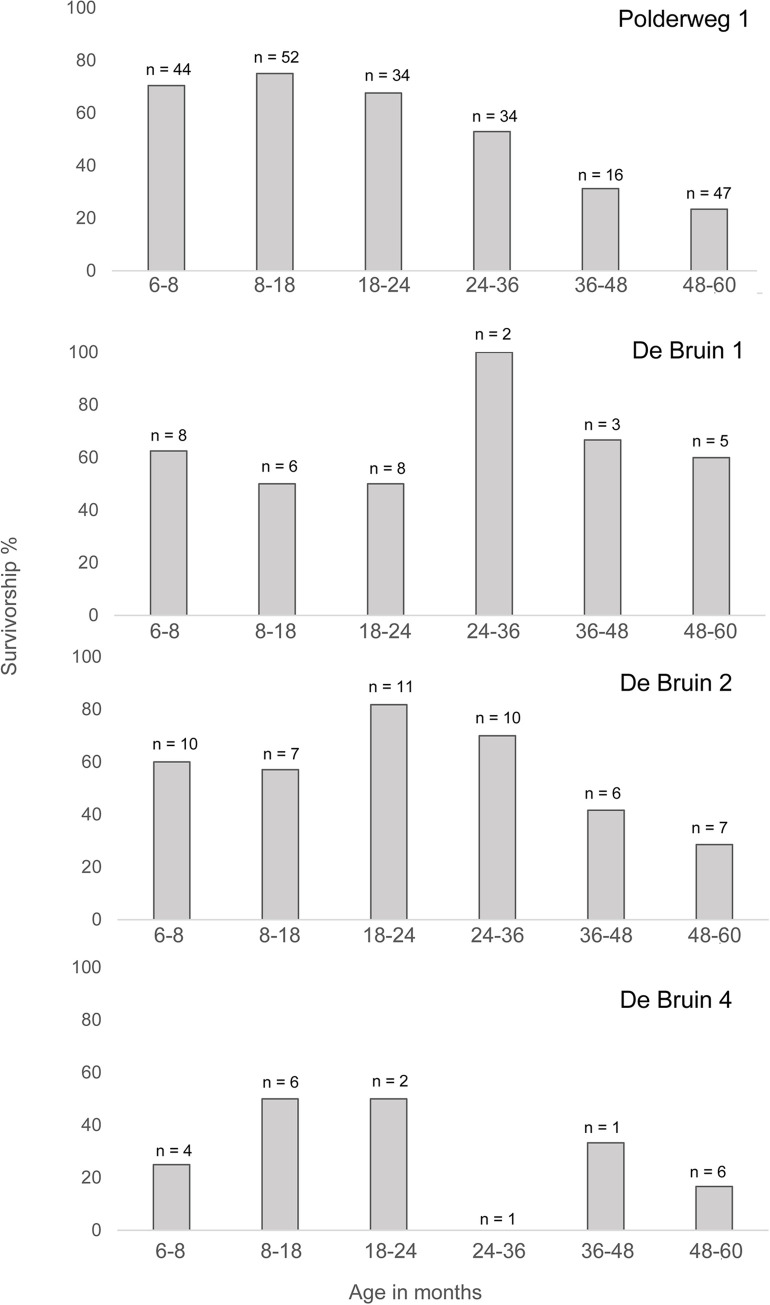
Survivorship profile based on percentage of fused long bones, for Polderweg Period 1 (n = 227), De Bruin Period 1 (n = 32), De Bruin Period 2 (n = 51), and De Bruin Period 4 (n = 22). Polderweg Period 2 and 3 had too few specimens to calculate the survivorship score. Survivorship percentage calculated following Zeder et al. 2015.

**Fig 5:**
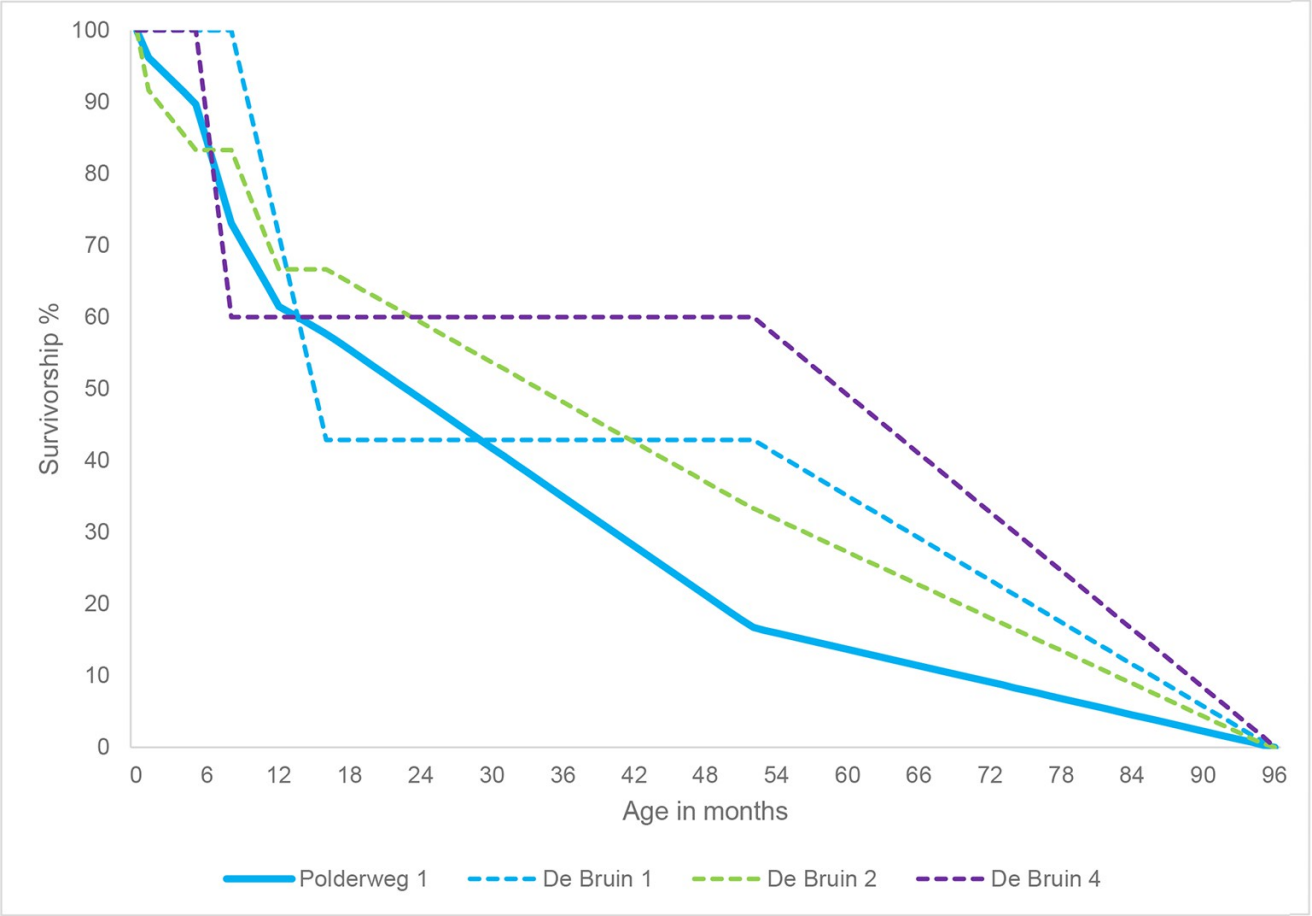
Survivorship profile based on tooth wear, for Polderweg period 1 (n = 39), De Bruin period 1 (n = 7), De Bruin period 2 (n = 6), and period 4 (n = 5). Period 3 has no specimens. Age classes calculated following Lemoine et al. 2014.

The lower teeth display a similar trend to the postcranial but not all differences are statistically significant (Fig 3; Fig 4 in S1 Fig). Smaller specimens are present at De Bruin 1 (mean = -0.022) than at Polderweg 1 (mean = -0.01) but the difference is not significant (Independent T-test, p = 0.201) (Table 14 in S1 Dataset). The LSI values of Period 2 at De Bruin scale close to Polderweg Period 1, with a mean of -0.005 and mean difference of only -0.005 (Independent T-test, p = 0.565). They do not differ significantly to Period 1 at the same site (Independent T-test, p = 0.214). There are only two specimens from Period 4, which scale very small (mean = -0.076). They are significantly smaller than De Bruin Period 2 (Independent T-test, p = 0.001) and Polderweg Period 1 (Independent T-test, p = 0.001). An ANOVA test showed there was significant difference between the three periods at De Bruin (p = 0.034). When the dental elements are compared for De Bruin as a whole versus Polderweg as a whole, De Bruin also scales smaller but not significantly so (Independent T-test, p = 0.192). The sample size of upper teeth from De Bruin is too small to allow intra-site or intra-period comparisons. However, overall Polderweg and De Bruin upper teeth show a similar trend as the lower teeth, except De Bruin 4, which has two larger individuals (Fig 5 in S1 Fig).

**Fig 6:**
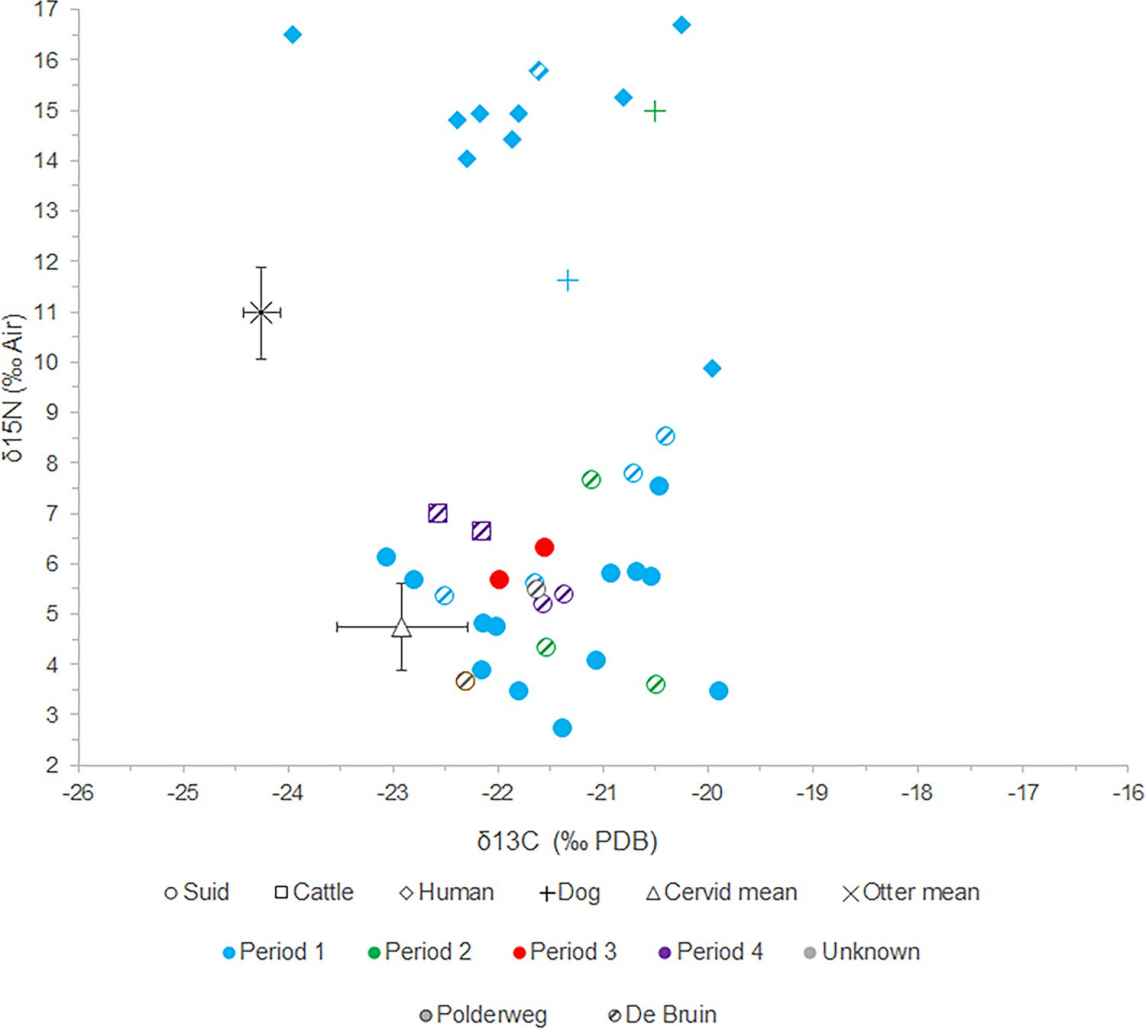
Scatterplot showing δ^13^C and δ^15^N values of suids from Polderweg (solid) and De Buin (pattern) per occupational period. Cervid mean represents the average of red deer and roe deer. The error bars displayed for the cervid mean and otter mean represent the standard deviations of these groups. Data on dogs and humans from Smits and van der Plicht [69].

To evaluate whether age of the individuals may be a factor in the significant postcranial size difference between the sites and periods, we reanalysed the data excluding two measurements, other than the scapula, that studies have shown to be age-dependent: the breadth of the proximal radius and the shaft width of long bones (Table 15 in S1 Dataset). The specimens from De Bruin 1 are still significantly smaller than Polderweg 1 (Independent T-test, p = 0.025) and those from De Bruin 4 are still significantly smaller than De Bruin 2 (Independent T-test, p = 0.011) (Fig 6 in S1 Fig). However, the difference between De Bruin 1 and 2 is no longer approaching significance (Independent T-test, p = 0.078). So, the measurements that are most age-dependent and least reliable for assessing size do not appear to be a determining factor in size difference between the sites and periods except for between De Bruin 1 and 2.

### Age estimation

Neither Polderweg nor De Bruin yielded identifiable postcranial remains belonging to neonates or 3–5-month-olds, or remains belonging to individuals older than 60 months (Table 11 in S1 Dataset). A couple of possible neonate specimens and individuals aged 3–5 months old were identified using tooth wear at both sites; there were no identified individuals older than 96 months (Table 9 in S1 Dataset). This suggests that very young piglets and old adults were in general not exploited. Although juvenile remains preserve more poorly than mature individuals, they tend to be well-represented in sieved assemblages. At Hardinxveld a substantial amount of the assemblage was obtained through sieving, including fish, bird, and botanical macroremains. It is therefore unlikely that the absence of juvenile remains is due to taphonomic processes.

The sample size of Polderweg Period 1 for the fusion-based age profile (n = 227) allows for a good reconstruction of the survivorship profile. It reveals that survivorship was highest for the youngest age classes: 6–8 months (piglets) and 8–18 months (yearlings) (Fig 4). After the latter age class, survivorship steadily declines with the biggest drop (20%) between the age classes 24–36 months (sub-adults) and 36–48 months (adults). Survivorship is lowest for the 48–60 months class (adults). The profile suggests that most suids at the site were killed in adulthood, between the ages of 18 and 60 months (1.5 and 5 years old).

The dentition-based age profile indicates highest mortality for individuals of 18–52 months old, followed by 6–8 months old, and subsequently 52–96 months old in Polderweg Period 1 (Fig 5). It thus reveals a slightly lower survivorship for piglets than epiphyseal fusion does. The sharp drop in survivorship for 18–52-month-olds (1.5–4.5 year-olds) is in line with the high exploitation of adults in this age group revealed by the fusion-based profile. The decline in survivorship of suids older than 52 months (4.5 years) shown by tooth wear is also in line with epiphyseal fusion. The lack of postcranial elements belonging to individuals older than 60 months might indicate that, of this oldest tooth wear age class, most individuals belong to around the minimum age (52 months) rather than the maximum age (96 months). It is important to note that the dentition-based age profile is based on a much smaller sample size (n = 39) than the fusion-based profile. However, studies have shown that a sample size of 30 or more specimens allows for the adequate reconstruction of the age profile of a population [48, 89]. Furthermore, the correspondence between the two profiles with regards to the emphasis on adult harvesting lends weight to their accuracy. Periods 0, 2, and 3 yielded too few specimens to reconstruct the age profiles for these periods.

De Bruin 1, 2, and 4 have altogether much fewer ageable specimens than Polderweg 1. It is not possible to make any inferences about survivorship based on tooth wear. The fusion-based survivorship profiles provide better representation. At De Bruin 1 (n = 32), the survivorship rate is approximately 60% for piglets and 50% for yearlings, lower than at Polderweg 1 (Table 11 in S1 Dataset). Conversely, survivorship is higher in the age class 24–36 months. However, there are only two specimens in this age class and overall, there is only a small number of specimens in each age class for De Bruin 1. This trend may therefore be more due to other factors than actual survivorship, such as taphonomic processes [cf. 47]. The survivorship profile therefore needs to be interpreted cautiously.

Period 2 at De Bruin yielded more postcranial elements (n = 51), allowing for a better reconstruction of the age profile. In this period, survivorship is lower for the age classes 6–8 months and 8–18 months than at Polderweg Period 1 with percentages of 60% and 57%, respectively. Conversely, there is a higher survivorship for the age class 18–24 months, namely 82% versus 68% at Polderweg. This could indicate higher survivorship for adult suids than at Polderweg or, considering the small dataset, it could be a result of taphonomic processes. Like at Polderweg, survivorship declines steeply for the age classes 36–48 months and subsequently 48–60 months.

De Bruin’s last occupational phase, Period 4, has only 22 specimens so this age profile must also be observed with caution. Survivorship is fairly low (≤50%) across the age classes. The profile is similar to De Bruin 2 but with even lower survivorship (25%) for piglets. This could suggest a comparable harvesting profile with possibly more emphasis on juveniles. Notably, neither the fusion-based or dentition-based analysis yielded any individuals aged 5 months or younger, thus piglets of suckling age, in this period.

### Stable isotope analysis

The results of the stable isotope analysis reveal clear similarities in the δ^13^C and δ^15^N values of suids from Polderweg and De Bruin and no apparent variations through time (Fig 6; Table 16 in S1 Dataset). The suids display a wide range of δ^13^C and δ^15^N values, between -23.06‰ and -19.89‰ for δ^13^C and between +2.72‰ and +8.53‰. for δ^15^N values. The mean values of suids from Polderweg 1, which provided the most robust sample size, are for δ^13^C -21.49 ± 0.87‰ and for δ^15^N +4.53 ± 1.09‰ (mean ± 1σ). One of the individuals from De Bruin 4 (EDAN0077) was identified as domestic based on size by the original report [9]. It is a fragment of mandible so we were not able to measure it to re-assess size. Its δ^13^C and δ^15^N values do not deviate from the rest. Four of the suids have elevated δ^15^N values compared to the rest but do not approach the δ^15^N ratios of the otters, dogs, and humans.

**Fig 7:**
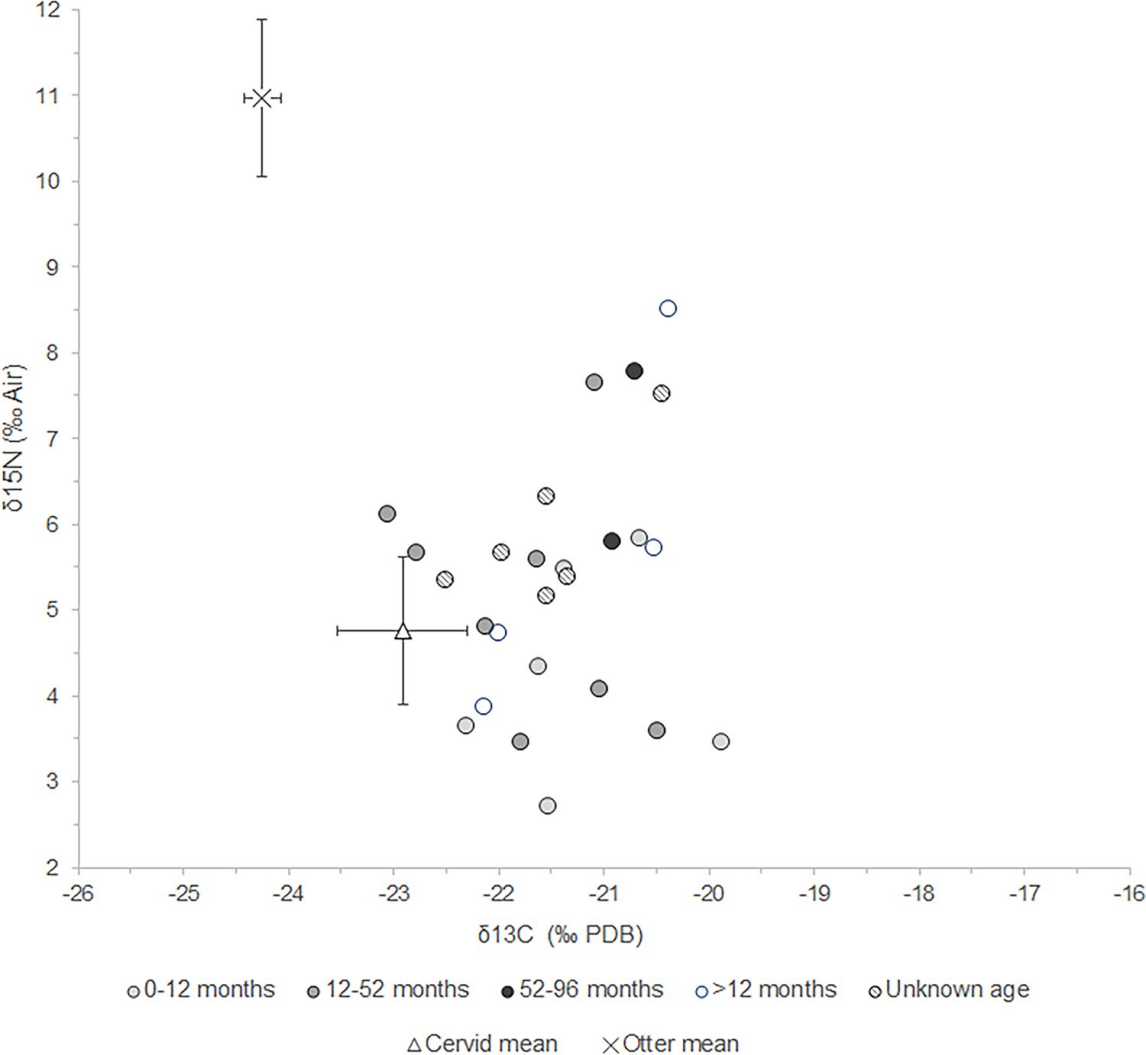
Scatterplot showing δ^13^C and δ^15^N values of suids per age category for both sites combined.

The suids’ range in δ^13^C and δ^15^N values is consistent across age classes, with yearlings, adults, and old adults not plotting markedly different (Fig 7). There are also no correlations between stable isotope values and LSI (Table 16 in S1 Dataset). The suids that scale larger in terms of LSI do not exhibit markedly different values than those that scale small. We were, however, not able to measure all of the sampled individuals, including the two suids from De Bruin 4, which in terms of context has the most potential to contain domestic individuals.

**Fig 8:**
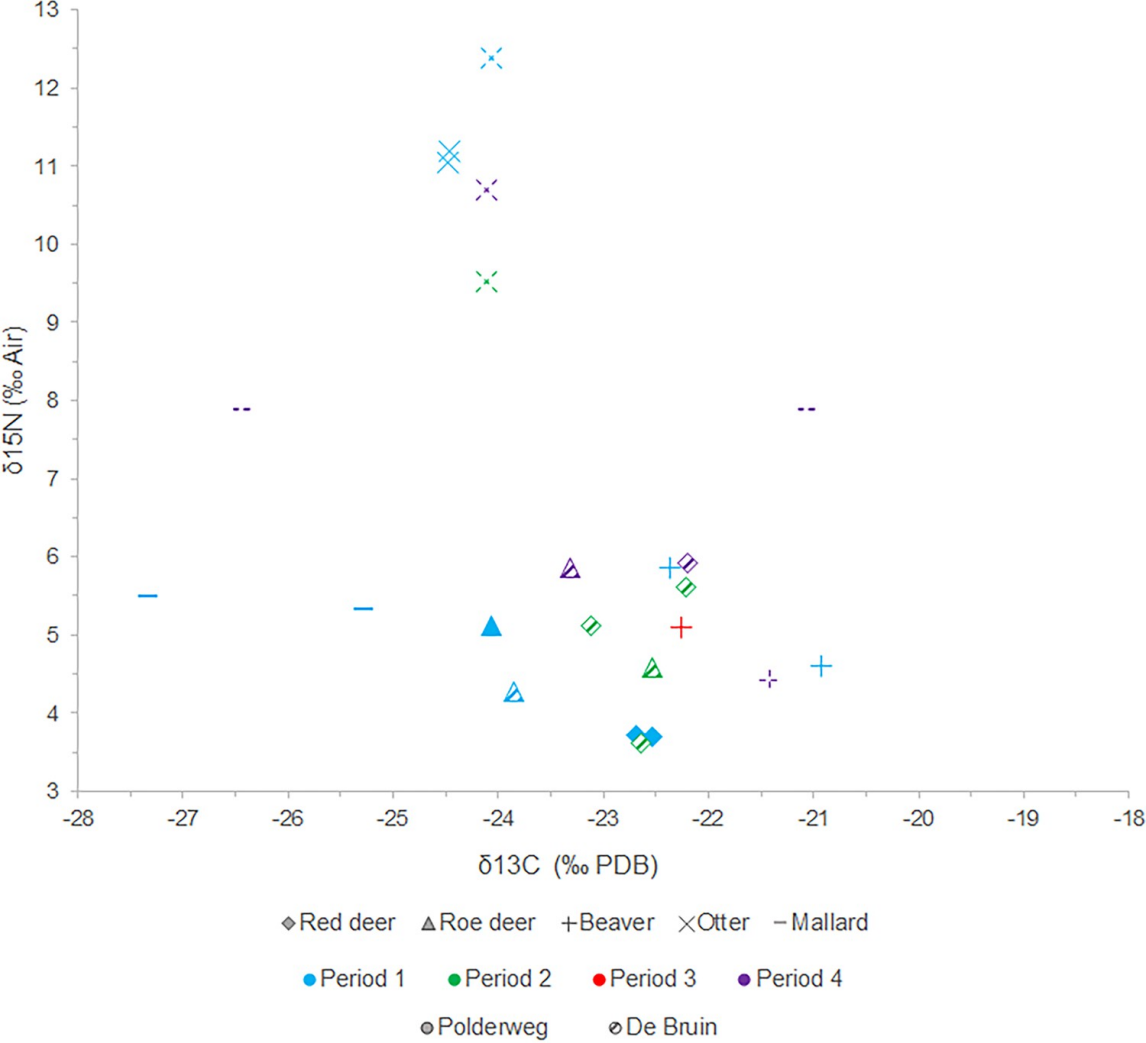
Scatterplot showing δ^13^C and δ^15^N values of wild fauna from Polderweg (solid) and De Bruin (pattern and dashed) per occupational period.

The suids display much more variation than the herbivores and in general less depleted δ^13^C values than the cervids (Fig 8). The roe deer and red deer plot fairly consistently with regards to their δ^13^C values; two roe deer have slightly depleted values compared to the rest. The range of δ^15^N values is in line with expectations for cervids [e.g. 80]. The two cervids from Period 4 have slightly higher δ^15^N ratios than the rest but they are within the margin of error. The two cattle specimens from De Bruin have slightly elevated δ^15^N values compared to the other herbivores. The otters display δ^13^C and δ^15^N values consistent with a freshwater fish diet and the beavers demonstrate isotopic compositions consistent with their niche as generalist herbivores. The mallards yielded a very wide range of values, which may be due to their diet as generalist foragers or because they are migratory, therefore not exhibiting local carbon and nitrogen signatures. Mallards therefore appear to be not well-suited as a dietary baseline.

## Discussion

The results of this study reveal that 1) suids were large in the Late Mesolithic and smaller suids are present in the Early Neolithic; 2) there is a probable shift in the harvesting profile over time; and 3) the suids exhibit a wide range of δ^13^C and δ^15^N values with no indications of differences through time, between sites, or between age groups. We discuss these results in relation to *Sus* palaeoecology, hunting strategies, and evidence for domesticated pigs.

### Suid size

The suids are large, clearly larger than at Schipluiden and Durrington Walls, until Period 4. The Late Mesolithic suids compare in size to other known Mesolithic wild boar in NW Europe. There is, however, also variation over time and between the sites, as shown by the postcranial elements and lower teeth (and to a certain extent, the upper teeth). For all periods except Polderweg 1, the sample sizes are small, necessitating caution when evaluating these results. The fact that the dental elements and postcranial elements display a similar trend lends weight to their representativeness. Furthermore, at both sites, the specimens come from different layers and segments, making it unlikely that the measurements reflect just one or a few individuals. Keeping the limitations in mind, we discuss some cautious interpretations of the results.

At Polderweg 1, the suids are large, scaling close in body size to the Scanian Mesolithic wild boar. The latter have been shown to have a large body size by modern standards [90]. Our results are consistent with findings by Albarella et al. [13]. They show that measurements of second and third lower molars and humeri from Polderweg plot in the upper range of modern European wild boar and close to measurements of Danish Mesolithic wild boar, which they demonstrate to have been among the largest in prehistoric Europe [13]. It is noteworthy that the Polderweg 1 suids scale smaller than the Scanian boar in terms of tooth size. However, dental elements and postcranial elements can scale differently and variations in postcranial and tooth size within one population have been documented [5, 90]. Based on the biometric results, the suids from Polderweg 1 are wild boar, which was expected for this Late Mesolithic occupation phase. The even distribution of the logarithmic indices, and thus lack of skewness, could reflect a fairly even sex ratio in the assemblage.

The only two measurable suids from the subsequent Period 2 at Polderweg scale smaller but with only two specimens it is not possible to interpret the reason for this variation. In contrast, the suids from De Bruin 2 show little difference with Polderweg 1. The results indicate that they too are wild boar and that, other than age, there was little difference in the harvested population at Polderweg 1 and De Bruin 2. The small size of the De Bruin 1 suids is therefore curious. There was exchange between the Hardinxveld occupants and Linearbandkeramik farming communities at this time, exemplified by the presence of exotic flint artefacts at the Hardinxveld sites [91, 92]. Import of domestic animals (or butchered parts thereof) is therefore not inconceivable. This combined with a continuation of wild boar hunting could explain the wide side range in this period. However, considering that the suids of De Bruin 2 again scale large, a different explanation seems more likely. We already endeavoured to correct for age (i.e., more juveniles in the assemblage). The difference between Polderweg 1 and De Bruin 1 is still statistically significant, suggesting that age is not a determining factor. A different sex ratio could instead be a cause. Wild boar are sexually dimorphic and postcranial elements have been demonstrated to be significantly affected by sex [39, 41]. Representation of sexed canines suggests that there is a high percentage of males in both site assemblages but this is likely due to a bias caused by artefacts. Osteometric methods that can help identify the sex ratio in the assemblages [39, 48] could not be carried out because the necessary measurements were obtainable only to a very limited extent. However, a different sex ratio is a viable explanation and the distribution of measurements for De Bruin 2 (Figs 3, 4 in S1 Fig) might suggest a tendency towards females. It could also explain why the dental elements scale smaller but not significantly so; tooth size is less affected by sex [41].

There is also evidence to suggest that hunting pressure can cause a size reduction in animals over time [93–95]. The survivorship profile of Polderweg 1 indicates a high adult mortality rate with few individuals surviving beyond the age of five years old. However, wild boar hunting appears to make up just a moderate part of the site’s hunting activities in this period, based on the proportion of wild boar (NF) in the mammalian assemblage (23%) (Table 1 in S1 Dataset). Therefore, wild boar do not appear to have been intensively exploited. Furthermore, it is unclear whether hunting pressure would affect tooth size in the same way as body size; in general this responds slower to selection pressure and other factors [41]. Yet the lower dental elements at De Bruin show the same trend in size fluctuation as the postcranial elements.

Climate has been demonstrated to have an effect on wild boar body size [13, 96]. There is, however, no evidence of climatic variation in the period 5450–4250 BC in the Netherlands, at least not on the scale observed to have an effect on size by Albarella et al. [13]. The palaeoenvironmental reconstruction of the river dune sites does indicate increasingly wet conditions over time and a decrease in forest cover but it is unlikely this would have been severe enough to affect the size of the highly adaptative wild boar. Magnell [90] has shown that despite the climatic change from the Boreal to the Atlantic period that affected vegetation and habitat in southern Scandinavia, wild boar did not decrease in size, probably owing to their omnivorous and opportunistic diet [90]. Furthermore, a study of linear enamel hypoplasia (LEH)–an indicator of nutritional and other stress factors–of Mesolithic and Neolithic European suids, which included specimens from Hardinxveld, demonstrated that the individuals from De Bruin and Polderweg had low levels of LEH [14], further underscoring that the population did not suffer from (nutritional) stress. It is therefore unlikely that hunting pressure or climatic changes can account for the variation in body size over time at De Bruin.

The size variation between Polderweg 1, De Bruin 1, and De Bruin 2 therefore is most likely caused by differences in the sex ratio of the harvested population. The small sample sizes of De Bruin, especially in Period 1, makes it difficult to determine the cause with certainty. It also remains possible that multiple factors were in play. The results suggest that it is unlikely that domestic pigs were already present in the period 5450–4800 BC.

This may have been the case though for Period 4, when the suids scale substantially smaller than at Polderweg 1 and De Bruin 2, both in body and tooth size. The sample size is very small but the differences are significant. The unfused postcranial elements also scale much smaller than those in the previous periods. The De Bruin 4 suids scale close to the suids at Schipluiden, a year-round occupied farming settlement dating to 3600–3300 where pig husbandry is more firmly established [45]. They are not, however, as small as the later Neolithic domestic pigs of Durrington Walls. There is nothing to suggest that the specimens are small due to age; excluding the most age dependent elements, the differences remain significant. It is possible that the small De Bruin 4 individuals might be mostly females; it is difficult to discount this option fully. Climate and hunting pressure are unlikely to have been a factor, as discussed above. It is therefore plausible that the remains include a substantial percentage of remains belonging to domestic pigs, which is not unlikely considering the presence of other domesticated livestock (sheep/goat) here in this period. If the specimens are domestic, it would explain why they scale close to the Schipluiden pigs. Equally, they could be hybrids. The small size of the dataset means it is not possible to discern whether there was a domestic pig population or just (butchered parts of) a few individuals.

### Harvesting profile

The harvesting strategy can be most accurately reconstructed for Polderweg 1 due to sample size and information from both fusion and tooth wear. Based on the results of the biometric analysis, the suids from this period are wild boar and harvesting can thus be discussed in terms of hunting strategies. Comparing the fusion-based survivorship profiles of De Bruin 1, 2, and 4 to Polderweg 1 suggests that the harvesting strategy may have changed. However, for all De Bruin periods the datasets are small, meaning we can interpret this shift only very cautiously. The datasets for Polderweg 2 and 3 are too small to reconstruct the survivorship profile. It is clear that the fusion-based analysis has advantages for the investigation of these sites due to the larger dataset and more detail in the adult age classes when compared to tooth wear [cf. 47].

Reviewing the fusion-based and dentition-based survivorship profiles of Polderweg 1, it is apparent that there was an emphasis on harvesting adult suids, aged between 1.5 and 5 years old (Figs 4 and 5). The rapid decline in survivorship through these age classes indicates that adults were targeted. Piglets and yearlings had a relatively high survivorship rate, although the dentition-based profile suggests some hunting of piglets. There appears to be little to no hunting of neonates and older adults. Modern wild boar populations are composed mostly of juveniles [97, 98]. The Polderweg assemblage thus does not reflect the natural demography of the population, implying a selective hunting strategy. This strategy does not appear to have been linked to seasonality of occupation. In Period 1, Polderweg was presumably a winter base camp. Yet hunting adults at the end of summer or in early autumn would result in a much greater yield because wild boar gain a significant amount of weight–and thus meat and fat–between late winter and late summer [99]. Furthermore, in winter, juvenile wild boar instead would have been easily available because the wild boar birthing season generally falls in the spring with peak births in March-May and litters are typically large [100–102]. While juveniles are represented in the survivorship profiles, it is clear that the emphasis lay on the hunting of adults. This hunting strategy thus does not appear to have been associated with the season of occupation. It is, however, somewhat of a circular argument to connect hunting strategy to seasonality as the season of occupation is partially based on the age of hunted animals. Revision of the evidence for seasonality could provide new insights into this [e.g. 99].

A hunting strategy concentrated on adult wild boar suggests, despite the winter season, a focus on the greatest yield. This concentrates on optimisation of the short-term return on the hunt, the so-called optimal foraging theory [103]. This strategy may not just have been aimed at getting the best yield in terms of meat, fat, and hide but also large canines for artefact production [104]. Twenty artefacts crafted from, primarily male, wild boar canines were found at Polderweg 1 [105] and they are in general a common occurrence at contemporary sites in the Netherlands and beyond [8, 106, 107]. The strategy could, furthermore, be linked to the prestige of hunting large adult wild boar. A focus on adult boar is also attested at other Mesolithic sites, such as Ageröd and Bredasten, Sweden [48] and Friesack, Germany [108]. While producing the optimal yield, it is less sustainable in the long run with regards to the wild boar population [48, 109]. Adult animals have the greatest reproductive capacity so selective hunting of them could eventually deplete the population [95, 110]. It is important to note, however, that the sustainability of the system–or lack thereof–is highly dependent on how many animals were targeted per hunt [cf. 103], something which we cannot reconstruct accurately for Polderweg.

In comparison to Polderweg 1, at De Bruin 1 piglets and yearlings have a lower survivorship rate while adults have a higher survivorship rate. A similar phenomenon for the young age classes can be seen at De Bruin 2, where overall the sample size is bigger, allowing for a better comparison. Here survivorship rates are approximately 10% lower for 6–8 month-olds and 20% lower for 8–18 month olds than at Polderweg, while there is a comparably high harvesting of adults between 3 and 5 years old (Table 11 in S1 Dataset). This suggests a possible shift in hunting strategy between Polderweg 1 and De Bruin 2 to not just target adults but also exploit juveniles. A larger proportion of juveniles suggests sounders were hunted, which are generally easier to locate and kill than solitary adults [104]. In general, the small dataset for De Bruin 1, especially, and for De Bruin 2 make it difficult to determine exactly how the hunting strategy developed between 5450 and 4800 BC. However, the results suggest a shift over time.

For De Bruin 4, the age profile of the population is also complicated by the small dataset. Overall, survivorship for 6–8 month-olds appears to be even lower than at De Bruin 2, indicating further increased harvesting of juveniles. Adult survivorship rates are approximately the same. Due to the small sample size, it is not possible to identify suid management through the kill-off pattern. The potential presence of both hunted wild boar and domestic pigs at De Bruin in this period further complicates this. The lack of neonate bones and lack of dental elements from suids of suckling age could be an indication that suids were not structurally being kept and husbanded at the site.

### Diet and foraging habitat

Diet and foraging habitat of the suids can most accurately be reconstructed for Polderweg Period 1; this period yielded a representative sample size [cf. 111]. By using these results as a baseline, it is possible to contextualise the results from De Bruin and the other periods from Polderweg. Our study reveals that the suids from Polderweg 1 have a wide range of stable carbon and nitrogen isotope values, within which the individuals from Polderweg 3 and De Bruin are firmly situated. The range is in line with the varied diet of wild boar, which are omnivores and generalist feeders. In general, plant matter, including roots, seeds, fruit, forbs, and bulbs, constitutes about 90% of the diet of modern wild boar [112]. The δ^13^C values are consistent with a diet consisting of this C_3_ plant matter. Three of the sampled individuals have low enough δ^13^C values (≤-22.5‰) to be consistent with a diet significantly affected by the canopy effect, i.e. foraging primarily in the forest [cf. 67]. Similarly, the roe deer and one red deer have depleted δ^13^C values <-22.5‰, comparable to depleted carbon values observed in roe deer from this period in other regions, which have been interpreted as a result of feeding in a forested environment [67, 80]. Modern wild boar prefer foraging under canopy cover and in deciduous forests [113, 114]. Oak and beech-dominated woodlands are especially preferred, with acorns comprising a major food source [112, 113], which would have been available to the Mesolithic wild boar on the river dunes. This corresponds with the individuals exhibiting depleted values. The rest of the suids (23 individuals) have less depleted values, but this may not preclude habitation of a woodland environment, which is not always expressed in low δ^13^C values [115]. Further baseline isotopic work on Dutch material is needed to explore the result of the canopy effect on δ^13^C values in this region.

The range in δ^13^C values of the suids may reflect the varied landscape of the Rhine-Meuse delta. The Hardinxveld environment comprised a mixed and open, deciduous forest (including oak) on the river dunes, surrounding open wetlands, and drier woodlands nearby. Wild boar are excellent swimmers and have home ranges of around one to several square kilometres [116, 117], so it is likely that they made full use of the habitats on and surrounding the river dunes. Furthermore, wild boar exhibit seasonal foraging behaviour, often switching between rooting in woodlands in the autumn and winter to grazing in open environments in the spring and summer [118, 119]. This seasonal foraging behaviour may lead to a mixing of δ^13^C values.

The majority of the suids exhibit δ^15^N values that are consistent with a wild boar diet containing mainly plant matter in a terrestrial and freshwater environment. The range in values is, like the carbon, broad but this is likely also due to the varied nature of suid diet. Furthermore, domestic pigs vary in δ^15^N values depending on age and sex [70], which could apply to wild boar too. Although we could establish no clear correlations between age groups and stable isotope values, age and sex may be a source of further variation. Four individuals from Periods 1 and 2 have elevated δ^15^N results compared to the rest, in particular sample EDAN0086. Of the factors that can cause elevated δ^15^N values, suckling can be excluded; three of the individuals are older than 12 months and one (number 42123) is at least older than a piglet of weaning age (typically, three to four months). Salt marshes lie ca. 50 km to the west so it is possible that these animals came from a saline environment. None of the individuals exhibited signs of pathologies but nutritional stress remains a possibility. A different environmental origin or nutritional stress therefore cannot be excluded as causes, but considering that suids are omnivorous, the most likely explanation for the elevated δ^15^N values is dietary enrichment in animal protein. Animal matter typically constitutes only a small percentage of the wild boar diet but is consumed frequently [112]. Their elevated δ^15^N values could thus be caused by a natural diet containing more animal protein than the majority of the sampled suids. Another possible source of animal protein could be anthropogenic, i.e., human refuse. The human stable isotope results and the abundance of freshwater fish remains found at the site indicates a human diet composed significantly of freshwater fish [9, 12, 69]. Consuming human refuse containing freshwater fish remains, and animal remains in general, would elevate the δ^15^N values of the suids. This has been suggested as an explanation for enriched δ^15^N levels of suids at Early Neolithic Danish and Norwegian sites, possibly indicating early pig management [3, 78].

At Hardinxveld, the four suids with elevated δ^15^N values plot close to one of the humans, but overall lower than the dogs and humans at the sites. This could suggest they were not consuming human refuse or, alternatively, they were consuming small amounts of it. A study of the effect of marine protein in domestic pig bone collagen showed that, in pigs and piglets (but not sows), there is a linear relationship between the amount of marine protein consumed and the δ^15^N offset [70]. If this holds true for protein acquired from freshwater fish as well, it means that suids consuming human refuse containing these protein sources would have elevated δ^15^N values in proportion to the amount they consume.

The context of these suids (deriving from Periods 1 and 2), and zooarchaeological evidence suggests that these individuals were wild boar. Indeed, two of the individuals with elevated δ^15^N values measure large in terms of LSI, suggesting they are wild boar (Table 16 in S1 Dataset). Opportunistic foraging around human settlements is a well-documented strategy for modern wild boar [120]. It is possible that the human activity and the resulting refuse at Polderweg and De Bruin attracted wild boar, resulting in the consumption of higher amounts of animal protein. The attraction of wild boar to human settlement is indeed one of the arguments for a commensal pathway to domestication for suids [121]. This cannot be verified for the Hardinxveld sites but this possibility should be kept in mind when interpreting irregular stable isotope results, even for the suids from the oldest phases.

The suid results indicate no differences between Polderweg and De Bruin, through time, or age groups. It cannot be excluded that variations over time in particular were not picked up by this analysis due to the small sample size from De Bruin and Polderweg 2 and 3. However, the results point to no apparent differences. This is supported by the analysis of wild fauna and the archaeobotanical evidence, which indicate a similar environment for Polderweg and De Bruin and no drastic changes in the foraging environment.

The range in δ^13^C and δ^15^N values of the suids from Polderweg 1 fits with the natural variation in wild boar diet and foraging habitat. This supports the conclusion from the biometric analysis that, as expected for the period 5450–5300 BC, the suids from Polderweg 1 are wild boar. The suids from the succeeding periods at Polderweg and the three periods at De Bruin fit within the range of the Polderweg 1 results. This could suggest that all of the sampled suids are wild or at least not discernibly managed in their diet. The suids from Period 4, the most likely candidate for domestic pigs, do not diverge in their results from the Polderweg 1 suids. The caveat is that our small sample set may simply be missing the domestic pigs. Similarly, the two cattle samples from Period 4 are within the typical range of both aurochs and domestic cattle [cf. 81]; therefore, it is not possible to determine if they are wild or domestic/managed.

Similar wide ranges have been established in Mesolithic and Neolithic wild boar from other regions [3, 75, 78]. There are currently no other studies of Dutch archaeological wild boar samples with which to compare. Other than the study of human diet at Hardinxveld [69], there is only one other archaeological study that includes stable carbon and nitrogen isotopes of suids in the Netherlands: a study of cattle at Schipluiden [55]. However, the majority of the animals, including the red deer and suids, had elevated δ^15^N values, probably due to the saline environment of this coastal settlement [55]. This difference in environment with Hardinxveld means a comparison between suid diet at these sites is not possible. This also underlines the importance of this study as a baseline for reviewing wild boar and early domestic pig diet in the Netherlands.

### Human-suid interactions at Hardinxveld

The findings provide new insights into the suids in the Late Mesolithic and Early Neolithic at the river dune sites, with the most notable findings being the large size and wide range in dietary regimes of wild boar at Polderweg 1 and the appearance of possible domestic suids in De Bruin 4. The results also allow for the exploration of developments over this time span in this key period. They demonstrate the presence of hunted wild boar assemblages at Polderweg 1, De Bruin 2, and probably also at De Bruin 1, so between 5450 and 4850 BC. There is no clear evidence for domestic pigs or managed suids during this period. These findings are in line with earlier studies that revealed large individual measurements [13] and low frequency of stress markers [14] on Mesolithic Hardinxveld suids, other indications that they are wild boar. The wild boar at Polderweg 1 and De Bruin 2 were large by modern European standards, a result which provides an important baseline for prehistoric wild boar in this region. At Polderweg 1 (5450–5300 BC), adult wild boar were targeted and wild boar make up 23% of the mammal remains in this period, suggesting a moderate contribution to the hunting activities at the winter base camp. Similarly, at De Bruin 1 (5400–5150 BC) occupation is mainly focused on the winter months and wild boar hunting continues to be a contributing activity, with remains making up 36% of the mammal assemblage.

Something then changes in the use of the river dune and wild boar hunting during De Bruin 2 (5000–4850 BC) when compared to Polderweg 1. At De Bruin 2, the proportion of wild boar in the mammal assemblage decreases substantially, to 12% of the mammal remains, and the results suggest a possible shift from targeting adult wild boar to targeting more juveniles. The small size of the De Bruin 1 dataset makes it difficult to determine with confidence how this period figures into these changes but it is possible the shift already started at this time. It is notable that in the same period, local ceramic production starts and a shift takes place towards multi-seasonal occupation. Louwe Kooijmans [122] interprets the two latter developments as features of the Neolithisation process, whereby hunter-gatherer communities were becoming more sedentary. Changes in hunting practices with regards to large ungulates have previously been noted in the advent of the Neolithisation process [5, e.g. 123]. However, at Hardinxveld, the first known livestock are not introduced until at least 400 years later and there is no evidence to suggest these communities were on a linear path towards farming [cf. 109]. The increased focus on beaver and otter trapping suggests instead a shift in resource utilisation.

This may have been linked to the evolving environmental conditions. Trees and forest decreased from Period 1 on, making way for more alder carr and shrubs, and the surface area of the river dunes decreased [29, 30]. As a result, the wild boar population on and around the river dunes may have decreased or became more dispersed, as they are dependent on forest cover for shelter as well as food [cf. 113]. This could explain why the human occupants focused even more on the trapping of beaver and otter and less on wild boar. Alternatively, it could have been a deliberate choice to concentrate hunting on the smaller mammals that flourished in the new, wetter conditions.

Lastly, it is also important to consider the agency of the prey. Predator-prey relationships are dynamic with mutual responses to each other’s behaviour [124]. Various studies show that wild animal populations change their behaviour and habitat use in response to hunting by humans and the presence of humans in their habitat [125, 126]. Wild boar in particular demonstrate high plasticity in this regard [117, 127]. The changes in hunting practices at Hardinxveld may have been a response to changes in animal behaviour reacting to, for example, increased, multi-seasonal human presence in the area. Sex and age ratios of hunted animals have been shown in some instances to be more a result of animal behaviour than choices made by the hunters [128]. This line of thought requires more research but it is worthwhile to bear in mind the agency of hunted animals when reconstructing prehistoric harvesting strategies.

After the developments around the turn of the sixth millennium, suid remains are much scarcer at Polderweg and De Bruin. Period 3 yielded exceptionally few artefacts, which is unfortunate because it could have shed light on what was happening prior to the appearance of livestock in Period 4 at De Bruin. The small size of the suid measurements from De Bruin 4 suggests at least some of the 61 suid specimens may belong to domestic pigs. Alternatively, they could be hybrids, reflecting admixture between local wild boar and introduced/feral domestic pigs. Genetic investigation of the remains could provide further insights into this (Erven et al. forthcoming). The stable isotope results and survivorship profile did not provide any evidence for management of pigs. However, a few domestic individuals (or the parts thereof) could have been present and would not be surprising given the presence of sheep/goat, which suggests the De Bruin occupants were familiar with livestock–or at least the products thereof. Other ‘Neolithic traits’ from this period include continued multi-seasonal use of the river dune [9, 27] and the production of pottery [23], as well as the possible evidence for dairy processing in one of the ceramic vessels [129].

It is questionable whether the small suids (and the domestic sheep/goats) at De Bruin 4 reflect the earliest signs of local, managed animal husbandry. At a younger phase of the nearby site Brandwijk (ca. 3900–3800 BC) and at the site Swifterbant S4 (4300–4000 BC), the presence of domestic pigs have more confidently been established based on genetic and biometric evidence [8, 11]. If domestic, the pigs at De Bruin therefore fit in the timeline of the appearance of domestic/managed suids, or could potentially represent the oldest in the Netherlands north of the LBK zone, depending on their exact date. Direct dating of the small specimens will shed more light on this [16]. Yet the rest of the faunal evidence from De Bruin 4 indicates a continuation of a broad-spectrum economy with an emphasis on fishing, fowling, and the trapping of beavers and otters. A similar diversity in exploitation can be seen at Brandwijk and Swifterbant, and indeed throughout most of the Neolithic Netherlands [45, 130, 131]. It is noteworthy though that at De Bruin there is much more emphasis on beaver and otter than on suids, in contrast to Brandwijk and Swifterbant. Additionally, at Brandwijk there is evidence for porcine fats in the pottery, in contrast to De Bruin where–other than the one possible dairy vessel–all yielded evidence for the processing of freshwater fish and ruminants [129]. Furthermore, at Brandwijk, after 3900 BC the biometric evidence, mortality profile, and body part representation also suggest the possible presence of domestic cattle populations, evidence which could not be established for De Bruin 4 [11]. This could therefore indicate that the turning point in the transition to animal husbandry in the Rhine-Meuse delta did not occur until around 4000 BC and after De Bruin’s occupation ended.

Seen from a wider perspective, the occurrence of small numbers of domestic suids and other livestock in these slightly earlier contexts from 4450–4250 BC, well prior to the emergence of local animal husbandry, fits in the current picture from northern Germany and southern Scandinavia. There too, domestic livestock specimens have incidentally–and sometimes tenuously–been identified in Late Ertebølle contexts, pre-dating the emergence of evidence for local animal husbandry around 4000 BC [132]. These are interpreted as arriving there through exchange with farming communities or even as escaped/stolen animals [132]. This is a credible explanation for the sheep/goat and possible domestic pigs at De Bruin. Other early finds of domestic livestock in Swifterbant assemblages correspond with this too [133]. The final period of De Bruin occupation therefore remains an enigmatic piece of the puzzle. It could represent a transitionary phase leading up to animal husbandry or it could instead reflect a new form of engagement by foragers with domestic animals through the consumption of their remains. Or it could mean a change in the behaviour of wild boar themselves, in response to the changing anthropogenic environment and landscape.

## Conclusion

With this multi-proxy study, we set out to explore human-suid interactions in the period prior to and leading up to the emergence of animal husbandry using the large faunal assemblages of the Hardinxveld sites. Our aim was to investigate wild boar palaeoecology and harvesting strategies, to uncover whether there is evidence for domesticated pigs, and to create a baseline of wild boar size and diet that can be used to further investigate the emergence of pig husbandry in this part of Northwest Europe. Our overall study were complicated by the small sample sizes available from the assemblages post-5200 BC. Nonetheless, the analyses provided significant results and new insights that can be summarised in three major conclusions.

First, in the period 5450–4850 BC, wild boar were hunted at the river dune sites and there may have been variation in the harvesting strategy over time. Between 5450 and 5300 BC, the foraging communities at Polderweg focused hunting on wild boar, beaver, and otter, as they did at De Bruin between 5400 and 5150 BC. At Polderweg, adult wild boar were targeted. In the following period 5000–4850 BC, at De Bruin there appears to be an increase in hunting of piglets and yearlings. Simultaneously, wild boar remains decrease in the assemblage and beaver and otter remains increase, suggesting less focus on wild boar hunting. These developments occur at a time when De Bruin witnesses occupation in multiple seasons and the start of local ceramic production. Hunting practices may have changed as an adaptation to increasing marshland, due to the more sedentary lifestyle, and/or in response to a potential shift in wild boar behaviour. The latter avenue requires more research but we propose that the agency of wild animals should also be considered as a potential factor affecting anthropogenic behaviour and changes in practices.

Second, suid exploitation in the final occupation phase at De Bruin, between 4450 and 4250 BC, involved a population of relatively small individuals. The small sample size from this period does not allow us to identify them as domesticated, female or hybrid individuals with any certainty, but the presence of domestic pigs would be in line with the appearance of sheep/goat remains at De Bruin in this period. However, the stable isotope results did not provide evidence for management of diet for the suids or cattle in this period. The lack of neonate and very young piglet remains could suggest that, if domestic, the pigs were not husbanded but instead originated from (gift) exchange with farming communities, either as individual animals on the hoof or as butchered parts. The incidental finds of early domestic livestock specimens prior to the emergence of local animal husbandry are known from elsewhere in Northwest European forager contexts. The results of this study suggest that in the Rhine-Meuse delta region too, the turning point in the transition to animal husbandry did not occur until around 4000 BC, and thus after occupation at the Hardinxveld river dunes ended.

Lastly, the findings provide a baseline of wild boar diet and size with which the emergence of pig husbandry in the Netherlands can be further investigated. The variation in wild boar δ^13^C and δ^15^N values are consistent with an opportunistic diet of primarily C_3_ plant matter and with the varied (seasonal) foraging behaviour of wild boar, as well as the freshwater environment. This study makes clear that the wide possible range of stable isotope values of wild boar should be taken carefully into account when investigating pig domestication. That being said, the use of a baseline of wild boar stable isotope values and background fauna can aid significantly. This study has contributed important new data to this for the Dutch wetlands, allowing for critical comparisons in future work. This study also provides a biometric baseline with which to later compare domesticated populations. The wild boar from the period 5450–5300 BC and 5000–4850 BC were large by modern and Mesolithic European standards. However, similar to the variability in diet, there was also variability in size. Smaller wild boar occurred between 5400 and 5150 BC, underscoring the fact that there is not ‘one size’ of a wild population, even within one region. This is also important to bear in mind in investigations of early animal husbandry through biometry. With the baselines established in this study, we aim to continue to explore the transition from wild boar hunting to pig husbandry in the Netherlands. Continued investigation of suids in the Swifterbant culture holds great potential to further elucidate the role of these animals in the transition to farming in the Dutch wetlands.

## Supporting information

S1 Dataset Supporting data for faunal, biometric, age estimation, and stable isotope analyses. (XLSX)

S1 Fig Supporting figures for faunal and biometric analyses. (PDF)
