# Supplementary material for: Hunting before herding: A zooarchaeological and stable isotopic study of suids (Sus sp.) at Hardinxveld-Giessendam, the Netherlands (5450–4250 cal BC)
Source: PLoS One. 2022 Feb 2;17(2):e0262557. doi: 10.1371/journal.pone.0262557 (PMC8809594; doi:10.1371/journal.pone.0262557)
Supplement: S1 Fig — (PDF) [file pone.0262557.s002.pdf]

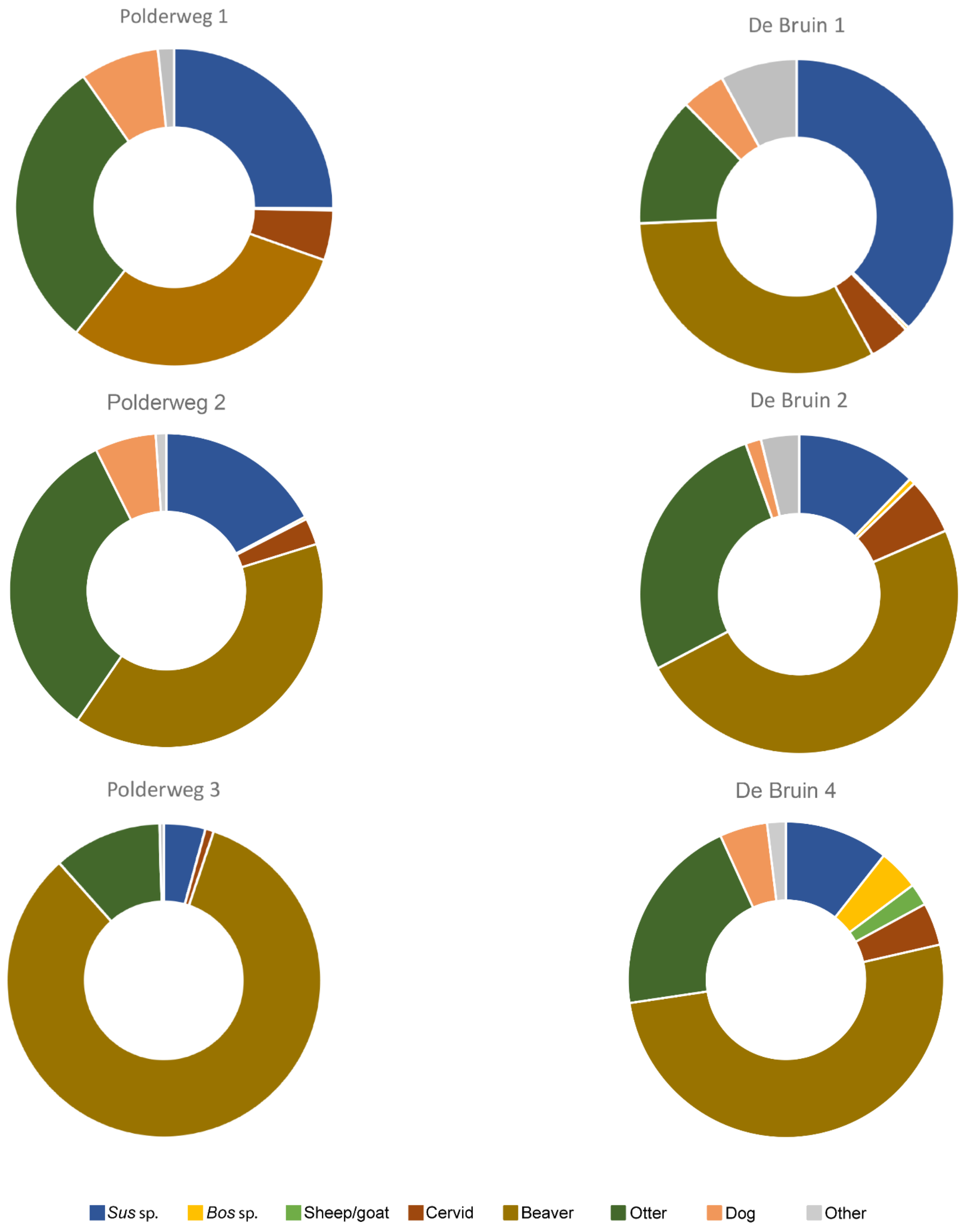

Figure 1 Number of Fragments of mammal species as a percentage of mammal remains per Period per site (data in Table 1 in S1 Dataset).

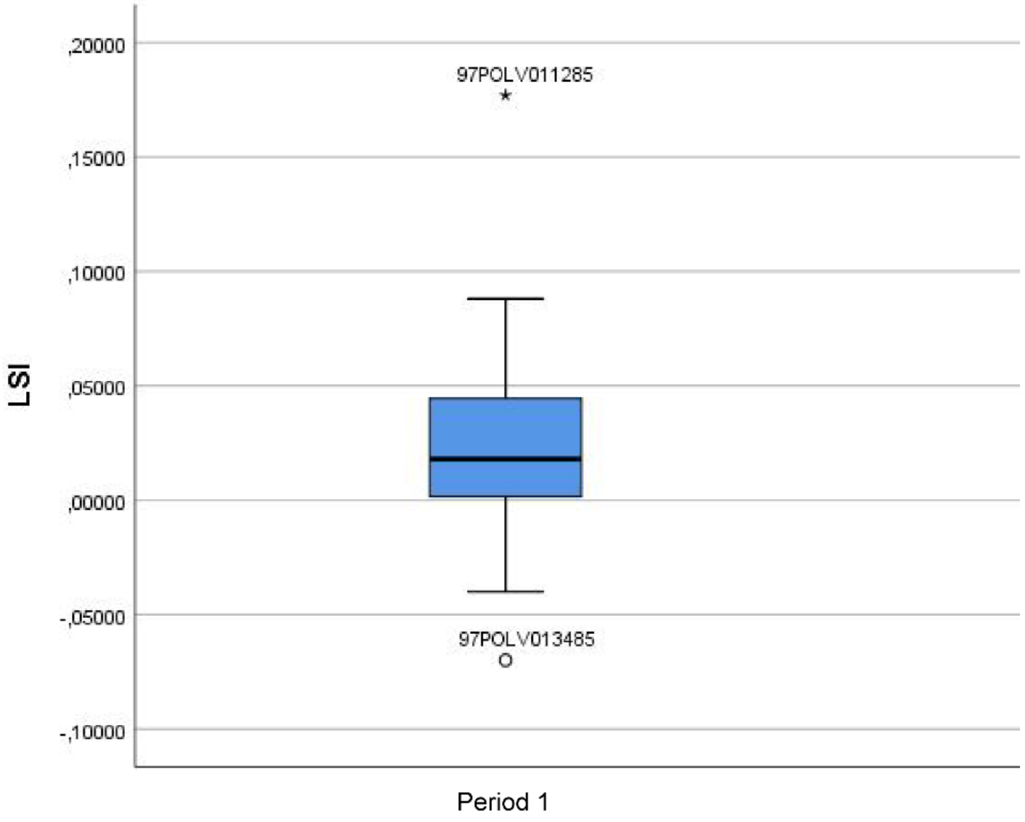

Figure 2 One extreme outlier in the Logarithmic Size Index (LSI) values of measurements on suid postcranial elements from Polderweg Period 1 (marked by *), identified as such as by a stem-and-leaf test, using IBM SPSS Statistics 26. Data in Table 5 in S1 Dataset.

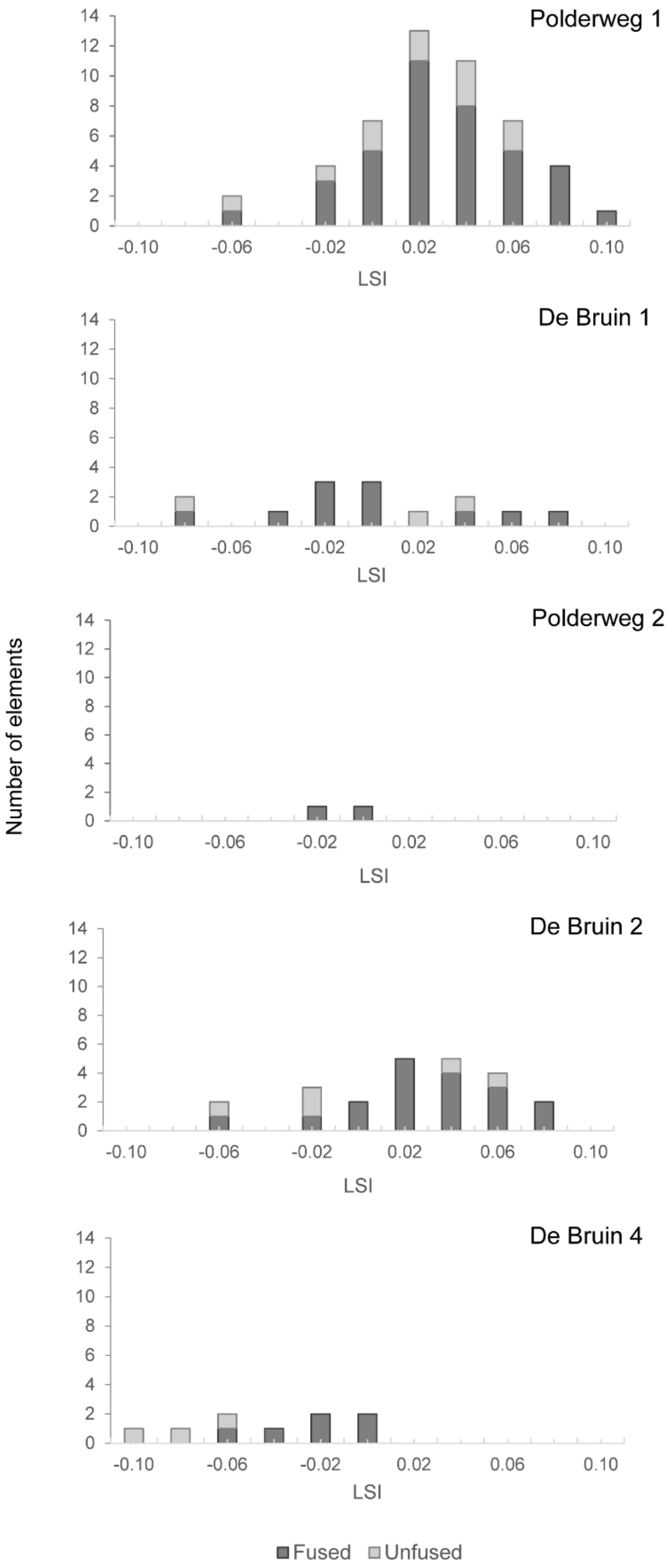

Figure 3 Histograms of Logarithmic Size Index (LSI) values of measurements on suid postcranial elements, per period and site: a) Polderweg Period 1 (n = 49), b) De Bruin Period 1 (n = 14), c) Polderweg Period 2 (n = 2), d) De Bruin Period 2 (n = 23), e) De Bruin Period 4 (n = 9). Metric data in Table 5 in S1 Dataset.

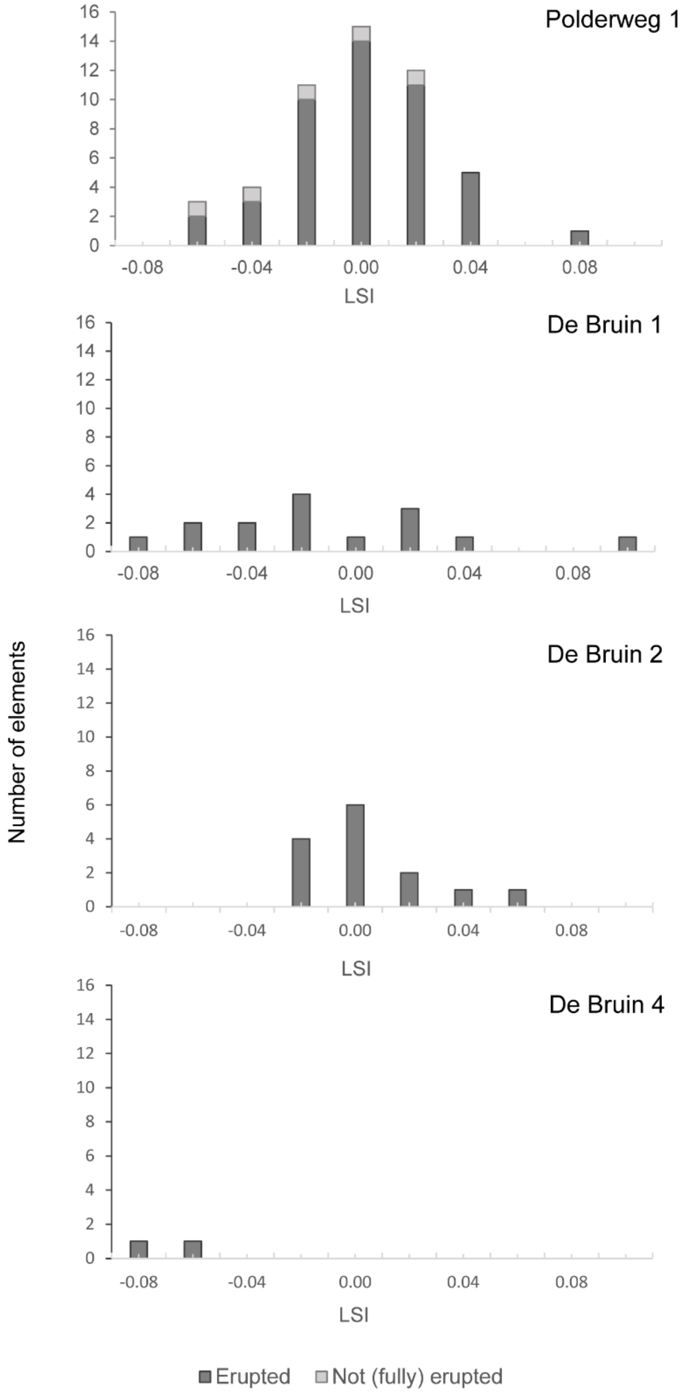

Figure 4 Histograms of Logarithmic Size Index (LSI) values of measurements on suid lower teeth, per period and site: a) Polderweg Period 1 (n = 51), b) De Bruin Period 1 (n = 15), c) De Bruin Period 2 (n = 14), e) De Bruin Period 4 (n = 2). Metric data in Table 6 in S1 Dataset.

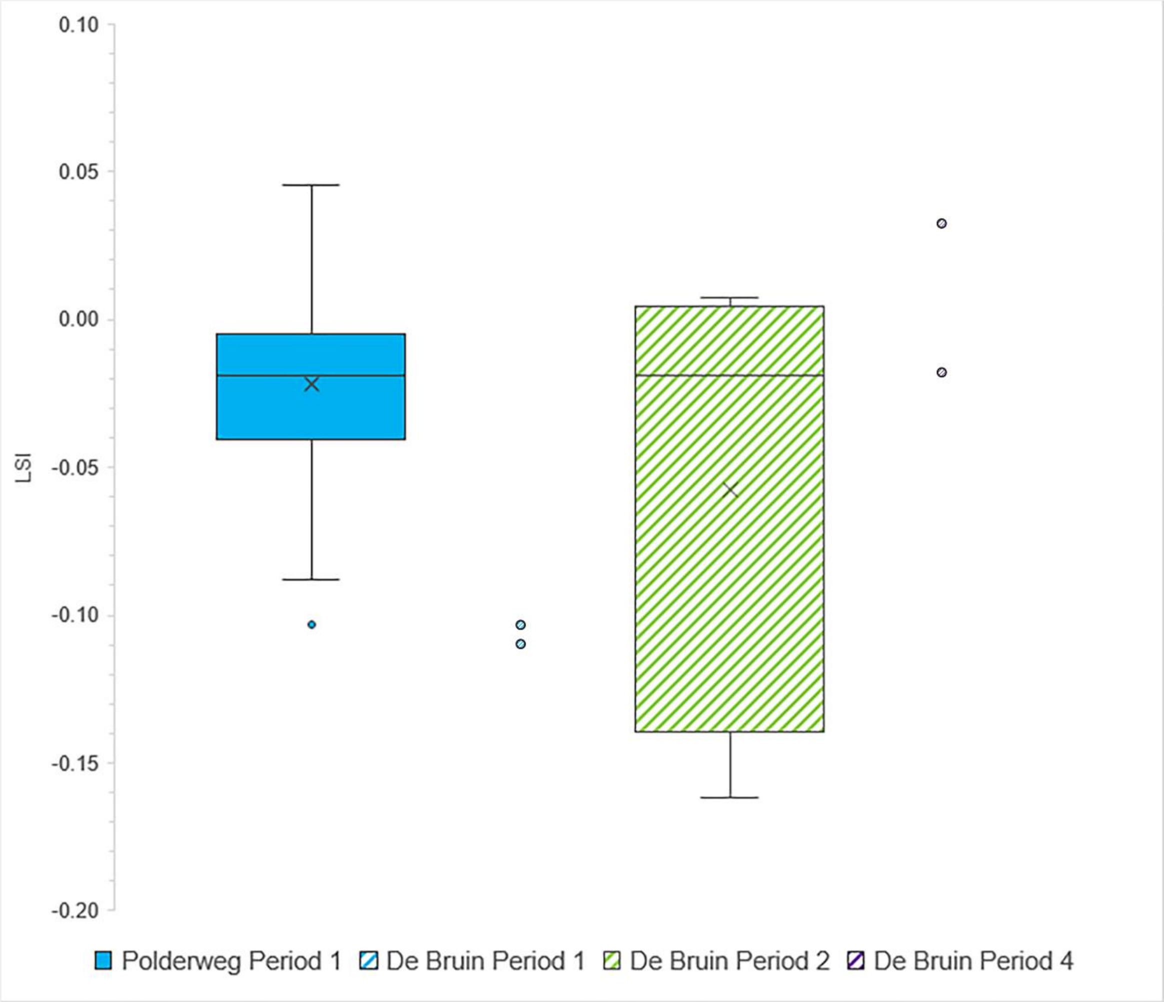

Figure 5 Boxplot comparing Logarithmic Size Index (LSI) values of measurements on fully erupted upper teeth from Polderweg period 1 (n = 49), De Bruin period 1 (n = 2; shown as two points), De Bruin period 2 (n = 5), and De Bruin period 4 (n = 2; shown as two points). Metric data in Table 6 in S1 Dataset.

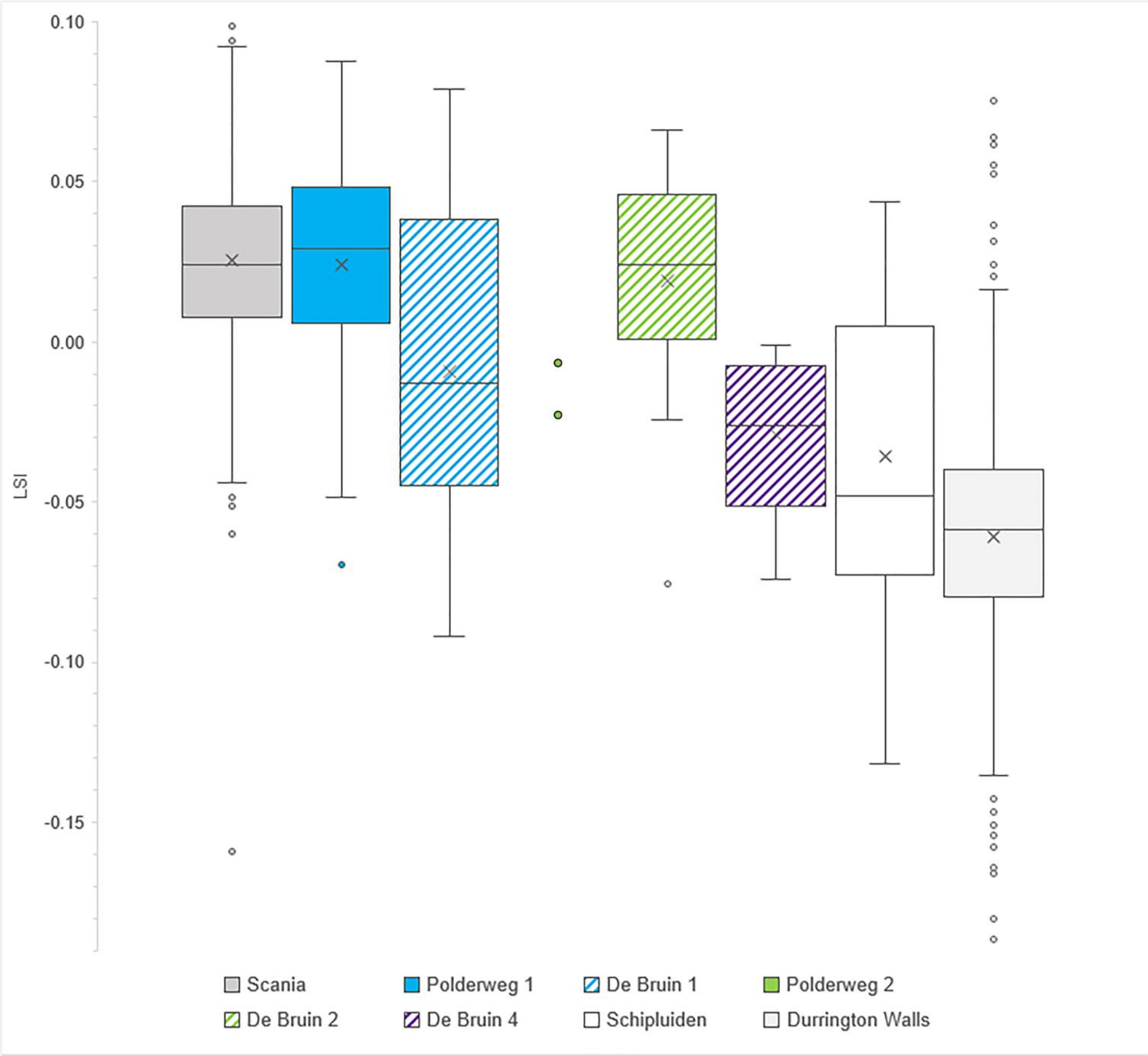

Figure 6 Boxplot of LSI values of fused postcranial elements, excluding proximal breadth of the radius and the width of the humerus shaft, comparing Scania (n = 324), Polderweg Period 1 (n = 30), De Bruin Period 1 (n = 9), Polderweg Period 2 (n = 2; shown as two points), De Bruin Period 2 (n = 17), De Bruin Period 4 (n = 5), Schipluiden (n = 17), and Durrington Walls (n = 663).
